# Contrasted evolutionary histories of two Toll-like receptors (*Tlr4* and *Tlr7*) in wild rodents (MURINAE)

**DOI:** 10.1186/1471-2148-13-194

**Published:** 2013-09-12

**Authors:** Alena Fornůsková, Michal Vinkler, Marie Pagès, Maxime Galan, Emmanuelle Jousselin, Frederique Cerqueira, Serge Morand, Nathalie Charbonnel, Josef Bryja, Jean-François Cosson

**Affiliations:** 1 Institute of Vertebrate Biology, Research Facility Studenec, Academy of Sciences, Prague, Czech Republic; 2 Department of Botany and Zoology, Faculty of Science, Masaryk University, Brno, Czech Republic; 3 INRA, UMR CBGP (INRA/IRD/Cirad/Montpellier SupAgro), Campus International de Baillarguet, CS 30016, 34988 Montferrier-sur-Lez Cedex, France; 4 Department of Zoology, Faculty of Science, Charles University in Prague, Prague, Czech Republic; 5 Laboratoire de génétique des microorganismes, Université de Liège, 4000 Liège, Belgique; 6 Labex CeMEB, Plateforme Génotypage-Séquençage, Université Montpellier2, Montpellier, France; 7 ISEM, Montpellier, France; 8 CIRAD, UR AGIRs, Montpellier, France

**Keywords:** Arms race, Host-pathogen interaction, Pattern recognition receptors, Adaptive evolution, Pathogen-Associated Molecular Pattern (PAMP)

## Abstract

**Background:**

In vertebrates, it has been repeatedly demonstrated that genes encoding proteins involved in pathogen-recognition by adaptive immunity (*e*.*g*. MHC) are subject to intensive diversifying selection. On the other hand, the role and the type of selection processes shaping the evolution of innate-immunity genes are currently far less clear. In this study we analysed the natural variation and the evolutionary processes acting on two genes involved in the innate-immunity recognition of Microbe-Associated Molecular Patterns (MAMPs).

**Results:**

We sequenced genes encoding Toll-like receptor 4 (*Tlr*4) and 7 (*Tlr*7), two of the key bacterial- and viral-sensing receptors of innate immunity, across 23 species within the subfamily Murinae. Although we have shown that the phylogeny of both *Tlr* genes is largely congruent with the phylogeny of rodents based on a comparably sized non-immune sequence dataset, we also identified several potentially important discrepancies. The sequence analyses revealed that major parts of both *Tlr*s are evolving under strong purifying selection, likely due to functional constraints. Yet, also several signatures of positive selection have been found in both genes, with more intense signal in the bacterial-sensing *Tlr*4 than in the viral-sensing *Tlr*7. 92% and 100% of sites evolving under positive selection in *Tlr*4 and *Tlr*7, respectively, were located in the extracellular domain. Directly in the Ligand-Binding Region (LBR) of TLR4 we identified two rapidly evolving amino acid residues and one site under positive selection, all three likely involved in species-specific recognition of lipopolysaccharide of gram-negative bacteria. In contrast, all putative sites of LBR_TLR7_ involved in the detection of viral nucleic acids were highly conserved across rodents. Interspecific differences in the predicted 3D-structure of the LBR of both *Tlr*s were not related to phylogenetic history, while analyses of protein charges clearly discriminated Rattini and Murini clades.

**Conclusions:**

In consequence of the constraints given by the receptor protein function purifying selection has been a dominant force in evolution of *Tlr*s. Nevertheless, our results show that episodic diversifying parasite-mediated selection has shaped the present species-specific variability in rodent *Tlr*s. The intensity of diversifying selection was higher in *Tlr*4 than in *Tlr*7, presumably due to structural properties of their ligands.

## Background

An effective immune defence is dependent on well-timed activation of an appropriate immune response. Pathogen recognition by innate immunity Pattern Recognition Receptors (PRRs) is crucial in this process [1,2]. The PRRs detect molecular structures named Microbe-Associated Molecular Patterns (MAMPs) that are conservatively present among individual microorganism taxa, because they are essential for their survival (such as, *e*.*g*., bacterial lipopolysaccharides, muramyl dipeptide, peptidoglycan, flagellin, mannose, bacterial, fungal, parasitic and viral nucleic acids) [3]. Recent studies have associated polymorphism in genes encoding PRRs with variability in resistance or susceptibility to several infectious diseases in humans, laboratory mice and poultry *e*.*g*. [4-8]. However, in wildlife, molecular variation in PRR genes is still poorly documented [9-14].

Understanding the evolution of the immune system in general has been a challenge for evolutionary biologists and ecologists since JBS Haldane associated natural selection with infectious diseases [15]. In vertebrates, the study of selection patterns was mostly oriented towards genes of acquired immunity which are now intensively studied even in wild populations. Among them, genes of the major histocompatibility complex (MHC) are the most explored and the role of balancing selection in their evolution is generally accepted and well understood [16-23]. The quite late discovery of genes involved in the second branch of vertebrate immunity, *i*.*e*. innate immunity, among which the most important PRRs are Toll-like receptors (hereafter abbreviated according to the mouse gene and protein nomenclature as *Tlr*s and TLRs, respectively) [24-27], has resulted in modest research of their evolution in wildlife populations [28].

Generally, two subclasses of TLRs are distinguished in vertebrates according to the ligands they target [3,9,29,30]. The first subclass includes TLR1, TLR2, TLR4, TLR5, TLR6 and TLR10. These TLRs predominantly detect bacterial components (but also fungal and to lesser extent viral components) and are expressed on the outer cell membrane. Throughout this paper we term them “bacterial-sensing” TLRs. The second subclass includes TLR3, TLR7, TLR8 and TLR9 and targets mainly viral components (*e*.*g*. ssRNA, dsRNA, DNA containing unmethylated CpG), hereafter termed “viral-sensing” TLRs. These TLRs are expressed mostly within cells into the membranes of endosomal compartments. This current spectrum of genes for TLRs arose by multiple gene duplication and during the last 700 Mya diversified to recognize distinct MAMPs [29,31-36].

TLRs of both subclasses are transmembrane proteins composed of three domains [34,37]. The Extra-Cellular Domain (ECD) consists of a varying number of Leucin-Rich Repeat motifs (LRRs) that form a horseshoe-shaped tertiary structure of the ECD. This domain contains the Ligand Binding Region (LBR) which is directly responsible for physical interactions with the pathogen-derived structures and as such it is likely subject to intensive selection. The ECD is followed by a short Transmembrane Domain (TM), and an Intracellular domain (ICD) containing the Toll/Interleukin-1 Receptor (TIR) domain responsible for TLR signaling [3]. As previously shown [38], non-synonymous SNPs located in LBR may affect the 3D structure of the protein and its surface charge. This may have important functional consequences, influencing receptor ability to bind pathogens [14,36,39], and may even lead to the evolution of species-specific ligand recognition [40,41]. Appropriate binding of MAMPs by LBR is connected with changes in receptor dimerization [42-44] that induce signaling and release of cytokines triggering mainly Th1 and Th17 inflammation, fever and phagocytosis [45-47]. The TLR signaling ensures an immediate response to invading microorganisms that, in a second step, further directs the following adaptive immune response [48,49].

Previous studies, mostly based on investigation in humans, primates and domestic or laboratory animals, provided information regarding some general patterns of TLR evolution and maintenance of their genetic polymorphism [2,9,50-52]. These studies revealed that the ECD is more frequently a target of positive selection than the TIR domain. Moreover, in general the viral-sensing TLRs seem to evolve under stronger purifying selection than the bacterial-sensing ones [53-56]. However, up to now, the evidence of TLR polymorphism and the type of selection that shapes this polymorphism in natural populations remain rare [10-14]. Besides, to our knowledge the precise investigation of the LBR variability and evolution is missing. Such information could nevertheless be important to better understand species-specific differences in the susceptibility to various pathogens [57].

In the present study we focused on the molecular variation of the genes encoding the bacterial-sensing TLR4 (binding mainly bacterial lipopolysaccharides, LPS, as a ligand) [58] and the viral-sensing TLR7 (binding viral ssRNA) [59,60] in 23 species of the subfamily Murinae. Murine rodents are largely distributed over the world and several species (such as rats and mice) live in close proximity to humans. A recent review showed that 60% of the agents of emerging diseases in humans circulate in animals [61] and most of the natural reservoirs of a number of serious viral and bacterial emerging agents of zoonoses are rodents [62,63]. Species-specific molecular variability in immune-related genes may be responsible for differences in the ability of rodent species to transmit these pathogens. Herein we aimed to document evolutionary histories of these two *Tlr*s during murine diversification. We implemented statistical approaches to infer *Tlr* phylogeny and to detect selection acting on DNA and amino acid (AA) sequences. We searched for deviations from “species” phylogeny based on a comparably sized non-immune sequence dataset by contrasting phylogenetic trees reconstructed from *Tlr* sequences with those reconstructed from “neutral” genes (both mitochondrial and nuclear). Deviations would indicate the occurrence of non-neutral patterns during the *Tlr* evolutionary history, *e*.*g*. adaptive selection [9,64,65]. Next we estimated putative functional changes in the LBR by examining variability in predicted tertiary 3D-structures of the proteins, and in biophysical properties of proteins (charge and structural characteristics) at polymorphic binding sites. Finally, we compared the evolutionary histories of the two TLRs to reveal potentially distinct evolutionary pressures shaping these proteins.

## Results

### Sequence analyses

Amplification and sequencing were successful in 96 samples representing 23 rodent species for *Tlr*4 and in 96 samples representing 22 species for *Tlr*7 (Additional file 1: Table S1). Only samples from one species - *Maxomys surifer* could not be completely sequenced for *Tlr*7 - the first 180 bp were missing and we excluded this species from the *Tlr*7 analyses. No stop codons, indels nor recombination were detected in these data using SBP (Datamonkey).

For the whole *Tlr*4 coding sequence (CDS), the three different domains were predicted by SMART as follows: ECD from AA position 1 to 635, TM from position 636 to 658 and ICD from position 659 to 835 in which the TIR domain (from position 671 to 816) and ICD distal part (ICD-DP; from 817 to 835) may be identified (Additional file 1: Figure S1). For *Tlr*7, the predicted location of the three domains was the following: ECD from position 1 to 850, TM from position 851 to 873 and ICD from position 874 to 1050 (TIR from 894 to 1033 and ICD-DP from 1034 to 1050; Additional file 1: Figure S1). In general, *Tlr*4 was more diverse than *Tlr*7, and within each *Tlr*, the ECD domain was more variable than the TIR domain in both molecules (Table 1). Surprisingly, ICD-DP located on the C-terminal end of *Tlr*4 represented the most variable region of exon 3 (π_ICD-DP-*Tlr*4_ = 0.102±0.015).

**Table 1:** **Estimates of sequence diversity and average codon**-**based evolutionary divergence over all sequence pairs for the exon 3 and particular domains of *Tlr*** **4 and *Tlr*** **7 genes**

***Tlr *** **domains**	***n***	***L***	**π±S.E.**	***hN***	***hA***	***S***	***Eta***	***dN±*** **S.E.**	***dS*** **±S.E.**	***dN/dS***
***Tlr*** **4**										
**Exon 3**	96	2247	0.049±0.003	122	90	545	625	0.038±0.003	0.102 ±0.008	**0.481**
**ECD**	96	1647	0.053±0.003	112	83	441	504	0.045±0.004	0.098 ±0.009	**0.597**
**LBR**	96	666	0.072±0.006	67	50	203	242	0.070±0.008	0.108±0.015	**0.787**
**TIR**	96	435	0.031±0.002	54	11	68	79	0.004±0.002	0.143 ±0.024	**0.067**
***Tlr*** **7**										
**Exon 3**	96	3147	0.034±0.003	79	49	466	518	0.021±0.002	0.088 ±0.007	**0.398**
**ECD**	96	2547	0.037±0.003	75	48	407	455	0.025±0.002	0.089±0.007	**0.468**
**LBR**	96	311	0.035±0.003	19	8	37	38	0.018±0.006	0.107±0.024	**0.196**
**TIR**	96	420	0.026±0.003	26	6	43	47	0.007±0.003	0.105±0.021	**0.070**

Note. - ***ECD*** extracellular domain, ***LBR*** - ligand biding region, ***TIR*** Toll/interleukin-1 receptor domain, ***n*** the number of sequenced individuals, ***L*** length of analysed sequences in base pairs, π average number of nucleotide differences per site between two sequences, ***S.*** ***E.*** Standard error, ***hN*** number of nucleotide alleles, ***hA*** number of amino acid variants, ***S*** number of polymorphic sites, ***Eta*** total number of mutations, ***dS*** number of synonymous substitutions per synonymous site (estimated by MEGA), ***dN*** number of non-synonymous substitutions per non-synonymous site (estimated by MEGA). Analyses were conducted using the Nei-Gojobori model; **S**.**E**. of *dN* and *dS* - were obtained by a bootstrap procedure (1000 replicates); ***dN***/***dS*** were computed by SLAC (Datamonkey).

### Phylogeny and co-divergence between the tree based on a comparably sized non-immune sequence dataset and TLR trees

Both phylogenetic approaches (MrBayes and RAxML) displayed similar trees for both *Tlr*s (Additional file 2: Figures S2 and S3). Minor differences between ML and Bayesian trees were found only at the intraspecific level. *Tlr*4 topology was well-supported with posterior probabilities (pp) ≥ 0.95 despite a lack of resolution within the black rat species complex (including *Rattus rattus*, *R*. *tanezumi*, *R*. *sakeratensis*, *R*. *tiomanicus*, *R*. *argentiventer*, *R*. *andamanensis*), between two *Bandicota* species (*Bandicota savilei* and *B*. *indica* did not form reciprocal monophyletic clades) and between two subspecies of the house mouse (Additional file 2: Figures S2a and S3a). Sequences of *Tlr*7 were also predominantly clustered according to species with strong supports (pp ≥ 0.95). Relationships between Asiatic mouse species were not fully resolved (monophyly of *Mus caroli*, *M*. *cooki* and *M*. *cervicolor* supported with a moderate pp value of 0.86 and Bootstrap values, Bp = 81) as well as those between *Leopoldamys* species (*L*. *edwardsi* appeared more closely related to *L*. *neilli*, rather than to *L*. *sabanus* but with a low pp of 0.6, Bp = 48). Similarly to *Tlr4*, branching orders within the genus *Rattus* were not resolved: *Rattus exulans* (clade I) was retrieved monophyletic without ambiguity (pp = 1, Bp = 100), *R*. *norvegicus* and *R*. *nitidus* were grouped together with the highest support (clade II, pp = 1, Bp = 100) and the remaining *Rattus* species formed a moderately supported group (clade III, pp = 0.7, Bp = 98, for more details see Additional file 2: Figures S2b and S3b).

**Figure 1:**
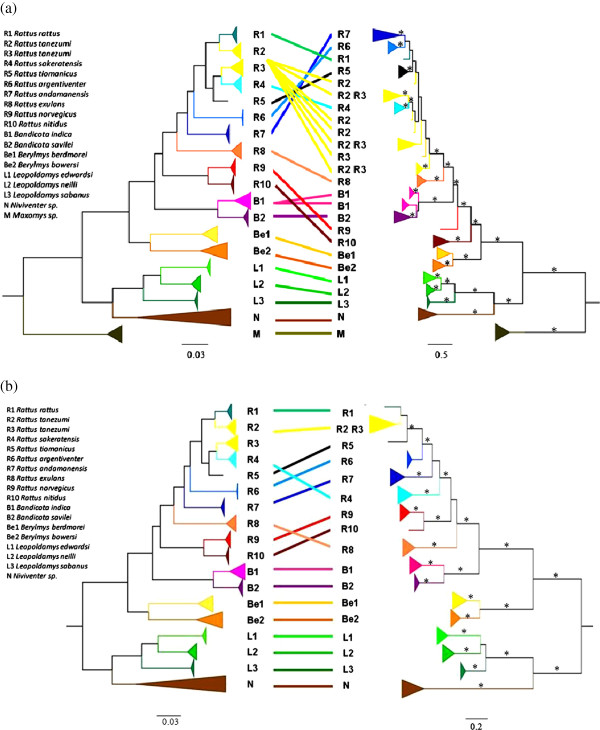
**Comparison of phylogenetic trees based on *Tlr*s and neutral markers.** Comparison of the Bayesian phylogenetic trees of *Tlr*4 **(a)** and *Tlr*7 **(b)** on the right with phylogenetic trees based on presumably neutral markers (*Cytb*, *Co*I, *Irbp*; for more details see Pagès et al. 2010) on the left. Abbreviations (R1, R2….M) indicate species assignment used in Pagès et al. 2010; corresponding legend is on the left. Color lines link the supported clades represented by the same species; * indicates posterior probabilities (pp) > 0.95.

At the first glance, *Tlr* phylogenies (based on MrBayes approach) of the black rat complex was congruent to the tree based on a comparably sized non-immune sequence dataset (Figure 1). The number of co-divergence events inferred using JANE 4 was significantly higher than expected by chance, meaning that the two phylogenies were similar (Additional file 1: Figure S4). However, the Shimodaire-Hasegawa test showed significant disagreement between the species tree and both *Tlr*s phylogenies (Δln L = 257, ddl = 1, *p* < 0.001 for *Tlr*4; Δln L = 76, ddl = 0.008, *p* < 0.05 for *Tlr*7), indicating that neither of the *Tlr* trees coincided precisely with the tree based on a comparably sized non-immune sequence dataset. The incongruence was mainly caused by recently diverged species of *Rattus*. However, we revealed several other differences, such as the misplacement of the genus *Bandicota* (occurring within *Rattus* in the *Tlr*4 tree) and the different positions of *R*. *sakeratensis* and *R*. *exulans* in species and *Tlr*7 trees (Figure 1).

### Evidence of signatures of selection

The comparison of ω (dN/dS) revealed substantial differences between the two *Tlr*s, as well as between gene parts encoding different domains (for details see Table 1). The difference between gene parts was mainly due to variations in the number of non-synonymous substitutions (which was higher in ECDs than in the TIR), while they both had similar numbers of synonymous substitutions.

**Figure 2:**
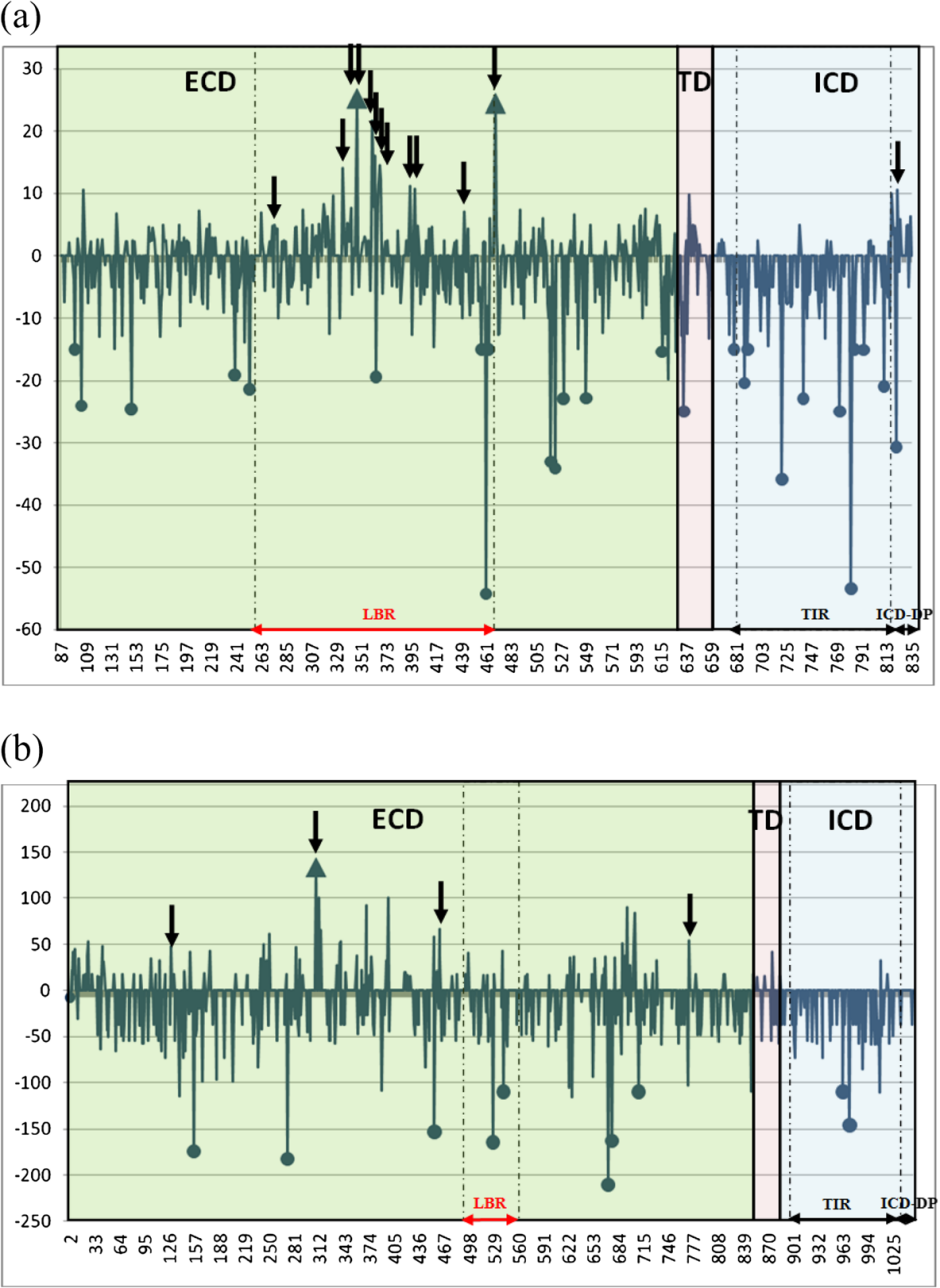
**Distribution of sites under selection identified by SLAC and MEME.** Intensity of selection acting on *Tlr*4 **(a)** and *Tlr*7 **(b)** exon3 with p < 0.05; the blue line is normalized dN-dS calculated in SLAC (Datamonkey); blue arrows-up - sites under positive selection detected by SLAC; black arrows-down - sites under positive selection detected by MEME (Datamonkey); blue full circles - sites under negative selection detected by SLAC. ECD - extracellular domain; LBR - ligand binding region; TD - transmembrane domain; TIR - TIR domain; ICD - intracellular domain; ICD-DP - distal part of intracellular domain.

The highly conservative SLAC (Single Likelihood Ancestor Counting) analysis (Datamonkey)[66] revealed two codon positions evolving under positive selection in *Tlr*4 and only one in *Tlr*7, all of them being located within the ECD domain (*p* < 0.05, Table 2, Figure 2). We found 26 and 10 negatively selected sites for *Tlr*4 and *Tlr*7 respectively (*p* < 0.05, Table 2, Figure 2), distributed evenly over the whole sequences.

**Table 2:** **Positively** (**MEME and SLAC**-**PS**) **and negatively** (**SLAC**-**NS**) **selected sites detected for the exon 3 of *Tlr*** **4 and *Tlr*** **7 at p** < **0**.**05**

	**ECD (and LBR)**	**TD**	**TIR**	**ICD-DP**
***Tlr*** **4**	**1**-**635 (248–** **469)**	**636-658**	**671-816**	**817-835**
**MEME**	273, 335, 345, 347, 361, 363, 366, 368, 394, 398, 442, 469	-	-	818
**SLAC**-PS	347, 469			
**SLAC**-NS	99, 105, 149, 240, 253, 364, 457, 461, 463, 518, 522, 529, 549, 616, 635	-	679, 688, 691, 721, 740, 772, 782, 785, 793, 811	822
***Tlr*** **7**	**1**-**850 (495** **–597)**	**851-873**	**894-1033**	**1034-1050**
**MEME**	128, 308, 461, 772	-	-	-
**SLAC**-PS	308			
**SLAC**-NS	156, 272, 455, 528, 541, 671, 676, 709,	-	963, 971	-

Note. - ***ECD*** extracellular domain, ***LBR*** ligand binding domain, ***TD*** transmembrane domain, ***TIR*** TIR domain, ***ICD-DP*** distal part of intracellular domain. Prediction of domains and numbering of sites are according to the reference protein sequence of *Rattus norvegicus* taken from GenBank [NP_062051.1 for *Tlr*4 and NP_001091051.1 for *Tlr*7]. Sites located in LBR are underlined.

The imprint of natural selection on protein coding gene is often difficult to reveal because selection is frequently episodic (*i*.*e*. it affects only a subset of lineages) [67]. We therefore looked for evidence of episodic diversifying selection at individual sites along the evolutionary branches of the trees using the MEME algorithm. Thirteen codon positions were found to be affected by episodic selection for *Tlr*4 (1.7% of all analysed codons) while only 4 codon positions showed this signature for *Tlr*7 (0.38% of all analysed codons). In *Tlr*4, 12 of these sites were located directly in LBR, while in *Tlr*7 none of the sites evolving under positive selection were in LBR. Whatever the *Tlr* gene considered, all sites found to evolve under positive selection using the SLAC were identified also by the MEME algorithm.

**Figure 3:**
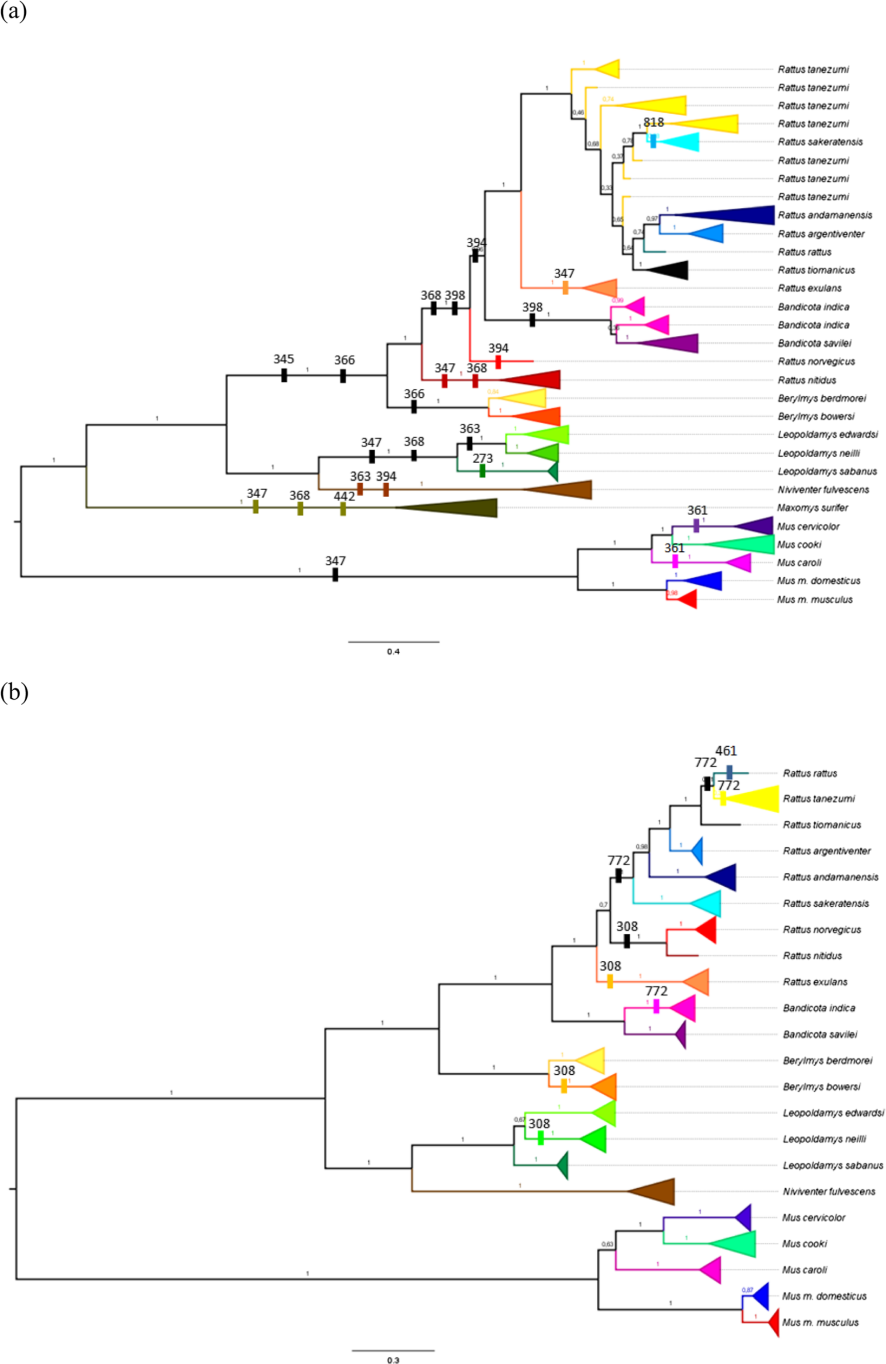
**Sites under positive selection identified in evolutionary lineages by MEME.***Tlr*4 **(a)**, *Tlr*7 **(b)** (significance level at *p* < 0.05), positively selected sites are marked and numbered above branches at simplify phylogeny based on MrBayes.

The signs of positive selection were scattered over whole *Tlr* trees, affecting nearly all branches of the *Tlr*4 phylogeny, both basal and terminal, while they mostly concerned the terminal branches for the *Tlr*7 phylogeny (Figure 3). Interestingly, one site evolving under positive selection (*p* < 0.05) was located in the ICD-DP of *Tlr*4 gene (Table 2, Figure 2a). We found that this part (*i*.*e*. the last 57 bp of C-terminal end of the protein following the TIR domain) was highly variable (19 nucleic acid alleles and 16 AA variants) with a mean ω = 1.11.

### Analysis of the ligand binding regions

**Figure 4:**
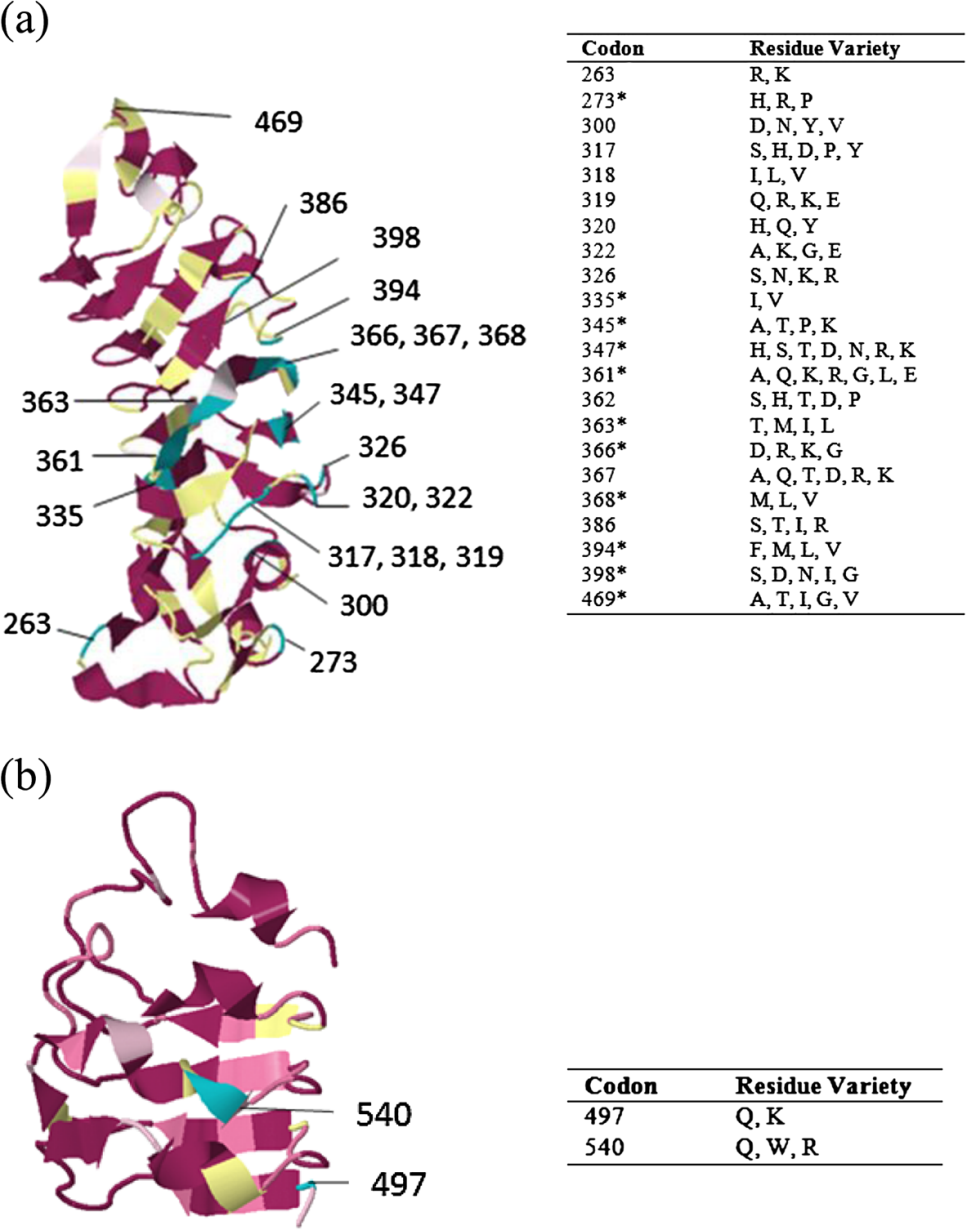
**Mapping of evolutionary conservation of amino acid positions in a protein molecule based on the phylogenetic relations between homologous sequences.** Conserved amino acid positions in LBR of TLR4 **(a)** and TLR7 **(b)**. Structure of LBR was analysed in Consurf; computations were based on MrBayes phylogenetic trees and tertiary protein structures of *R*. *norvegicus* [Gen Bank Acc. KC811688/ KC811786]; most variable positions are highlighted in turquoise and numbered (grade 1); most conserved sites are in violet; yellow sites mark insufficient data; white sites have average conservation score; tables show residue variants at the phylogenetically variable positions with grade 1; codons with asterisk have been identified as those under positive selection by MEME.

In general, the Ligand Binding Region (LBR) was much more variable in *Tlr*4 than in *Tlr*7 genes. We detected 50 different AA variants of the LBR in the TLR4 dataset, while only eight different AA variants were detected in TLR7. Out of the 222 AA sites of LBR_TLR4,_ 43% were polymorphic, while among the 103 AA sites of LBR_TLR7,_ only 10% exhibited genetic variations. The Consurf analysis performed to estimate the degree of evolutionary conservation of each amino acid position in LBR revealed 10% of phylogenetically variable positions (*i*.*e*. 22 positions assigned to grade 1 and corresponding to the most variable and rapidly evolving amino acid positions out of 222 positions in total) in TLR4, but only 2% (2 positions with grade 1 out of 103) in TLR7 (Figure 4). Other positions were assigned as conservative (57% and 79% in TLR4 and TLR7, respectively) or had insufficient support (33% and 19%, respectively; Figure 4).

Ligand-binding positions in rodents were predicted by comparison with those identified in humans by Park et al. [39]. In TLR4, two out of eight LPS-binding amino acid positions were identical to humans and strictly conserved among rodents (F438 and F461). Three other were conserved in terms of amino acid features (*i*.*e*. polarity, hydrophobicity) but distinct from human residue and variable among rodents (R263K, K360R and K434R). Interestingly, one LPS binding site that was uniform in human was found to be evolving under positive selection using the MEME algorithm. We found hydrophobic and hydrophilic residues, although this position, L442Y, is known to be involved in hydrophobic interactions. Finally, two remaining positions were found to be highly variable in rodents (339 and 386) (Additional file 1: Table S3). In TLR7, the nine ligand binding residues predicted following Wei et al. [68] were strictly conserved within rodents and seven of them were common to both rodents and human TLR7 (Additional file 1: Table S4).

The pairwise RMSD that allowed estimating the differences in 3D protein structure among variants varied from 0 to 1.5Å in TLR4 variants, and from 0.6 to 1.7Å in TLR7 variants (Additional file 1: Figure S5). Yet, in the phenetic diagram of TLR4, 3D-structures of *Rattus sakeratensis* and *Rattus nitidus* were distinct from each other and also from all other species. Similarly for TLR7, the 3D-structure of the protein of *Rattus exulans* was separated from other species (Additional file 1: Figure S5). To provide wider context we performed additional comparison between PDB structures (obtained from The RCSB Protein Data Bank http://www.rcsb.org/pdb/home/home.do) of human (HoSaTLR4-3fxi_A) and mouse (MuMuTLR4-3vq2_A) ECD_TLR4_ and between ECD of mouse TLR4 and TLR3 (MuMuTLR3-3ciy_A). The comparison between species of the same TLR was 1.7Å (HoSaTLR4-MuMuTLR4). Comparison between two TLRs from most distant TLR families of the same species was 4.6Å (MuMuTLR4-MuMuTLR3). The analysis of electric charge of LBR revealed higher variation in TLR4 (from −7.7 to 1.5) when compared with TLR7 (from −1.6 to 0.6). Detailed analyses of LBR_TLR4_ revealed that *Mus* and *Rattus* species were well differentiated from each other (*Mus*: from −7.7 to −3.7; *Rattus* and related genera: from −3 to 1.5, Additional file 1: Figure S6a). Similar pattern was found for LBR_TLR7_ (*Mus*: -1.6, *Rattus* and related genera: from −1.4 to 0.6, Additional file 1: Figure S6b).

## Discussion

In this study we analysed the variability of two important vertebrate immune genes involved in innate immunity across wild murine rodents and we looked for evidence of selection. Overall, we found that *Tlr*4 was much more variable than *Tlr*7 and that the evolution of both genes had been influenced mostly by purifying selection. However, comparison of both *Tlrs* revealed contrasting evolutionary patterns. *Tlr*7, which is involved in the recognition of viral nucleic acids, was highly conserved across rodents and its evolution seemed to be strongly shaped by purifying selection. Predicted ligand binding sites in LBR_TLR7_ were identical across all species and only few sites were detected to evolve under positive selection within the whole molecule. By contrast, *Tlr*4, which detects several different pathogen ligands, was more variable and was affected by numerous events of episodic selection. Positively selected sites mostly occurred in LBR, probably as a result of co-evolution with pathogens. Analyses of the LBR variability in surface charge revealed a potential for interspecific differences in ligand binding capacities of both *Tlr*s.

### Differences in TLRs evolution - phylogenetic approach

We found that both *Tlr*s were conserved genes as their phylogeny almost correctly recapitulated species phylogeny. In spite of this conservatism we revealed some incongruence between gene and species topologies, especially in branches represented by the shallow genealogy of the black rat complex and *Bandicota* spp. (Figure 1a). These species have experienced recent and rapid radiation during the Early Pleistocene about 1 Mya [69,70]. Discrepancies between a gene genealogy and the species phylogeny in recently diverged species often results from Incomplete Lineage Sorting (ILS) of ancestral polymorphism and/or episodic gene flow and hybridization [71,72]. Indeed, *R*. *tanezumi R2* and *R*. *tanezumi R3* were recently proposed as conspecifics or were suspected to hybridize in Southeast Asia [73]. In addition, hybridization with introgression occurred between the invasive populations of *R*. *tanezumi* and *R*. *rattus* in the United States [74]. These phenomena could explain incongruence between *Tlr*s and species trees. However, directional selection could also be involved. Discrepancies in *Tlr*7 phylogeny represented by *R*. *exulans* and *R*. *sakeratensis* seem more likely to be caused by pathogen selective pressure (Figure 1b). ILS and hybridization are unlikely to result in such deeper changes, whereas the influence of directional selection (positive or negative) on non-neutrally evolving genes could be at more likely explanation [75]. The rejection of co-divergence (concerning basal nodes) between *Tlr*s and species phylogenies could reflect the occurrence of pathogen-driven selection on *Tlr*s during the evolutionary history of the murine rodents [32,76]. The former hypothesis should now be tested by a detailed analysis of spectrum of pathogens from rodents to determine if the species producing the incongruent topology displayed specific pathogens that could mediate this selection.

### Tlr variability and signatures of selection

We found that 92% and 100% sites (respectively for *Tlr*4 and *Tlr*7) evolving under positive selection were located in the ECD, which is responsible for pathogen recognition. For *Tlr*4 92% of these positively selected sites found by MEME algorithm were located in the LBR. This is in concordance with several recent studies conducted on primates, birds and rodents, that have suggested a high accumulation of positively selected sites at LBR [9-11,77,78]. Surprisingly, none of the sites evolving under positive selection was identified directly in the LBR of *Tlr*7.

The TIR domain of both *Tlr*s was evolving under much stronger functional constraint than the ECD in both genes. We found only 11 amino acid variants of TIR_TLR4_ in 23 species and six different variants of TIR_TLR7_ in 22 species. Altogether our results support the observation that *Tlr* exodomains evolve more rapidly than the intracellular TIR domain [9,56,77,78]. The requirement of sites within ECD, which would be involved in ligand recognition and able to recognize permanently fast-evolving pathogens, could explain this pattern. Besides, the high conservation of the TIR domain could be adapted to maintain a functional response of signal transduction see, e.g. [9,33,50,56,58,79].

Both genes showed non-significant differences between ECD and TIR with respect to *dS*, supporting the hypothesis that there was no difference in mutation rate between ECD and TIR. The same result has been found in comparative studies of 10 vertebrate TLRs [33]. The distal part of ICD in *Tlr*4 was surprisingly highly variable among rodent species. The reason for such a high level of variability is still unknown; however some authors suggest that this region at the carboxy-terminal end of *Tlr*4 could be responsible for interspecific differences in LPS sensitivity [50].

Positive selection we also detected using the MEME approach that individually considers each codon along the *Tlr*s phylogeny [67]. We found that episodic positive selection affected most lineages in the phylogenetic tree of *Tlr*4, while the situation was quite different in *Tlr*7, where the sites evolving under positive selection were mostly distributed only along the terminal branches. Episodic diversifying selection could have affected *Tlr*4 throughout its evolution and this process could still be in operating, while in *Tlr*7 diversifying selection seemed to have appeared more recently and the gene history was mostly maintained by the stronger purifying selection (Figure 3).

### Analysis of the Ligand binding region

In TLR4 variants we found 22 rapidly evolving positions distributed all over the LBR. While TLR4 is able to detect several ligands, the most studied one is LPS of Gram negative bacteria. TLR4 does not interact with LPS alone directly but forms stable heterodimers with MD-2 [80]. Analysis of the crystallographic structure of mouse TLR4-MD-2-ligand complex has shown that the interactions between TLR4, -LPS and MD‒2 take place on the concave surface of TLR4 [80]. We predicted that sites involved in the TLR4-MD-2 interaction should be highly conserved to maintain the receptor function in LPS binding and these sites were thus not identified in the present study. Among the eight known LPS-binding sites, identified by Park et al. [39] in humans, two residues (F438 and F461) were conserved between humans and rodents as well as among rodents. These key residues are jointly involved also in hydrophobic interactions between TLR4 and MD-2 [39,81]. It is possible that negative selection might maintain an invariable combination at these sites to preserve MD-2 binding, which supports our hypothesis mentioned above. One exception was the controversial site L442Y which was suggested by Park et al. [39] to be also involved in hydrophobic interactions between TLR4 and MD-2, but Resman et al. [81] challenged the importance of its function. Among the studied rodents this codon was found to be polymorphic and has been shown to be affected by episodic positive selection during rodent evolution. A hydrophobic nonpolar residue (Leucine, L) was commonly shared between rodent species except for *Maxomys surifer* that harbored a hydrophobic and polar Tyrosine (Y). For three LPS-binding sites, R263K, K360R and K434R, the biochemical features of the residue were maintained between rodents (all were positively charged residues) but distinct amino acids were detected. The important role of these residues was supported also by Ohto et al. [82] and the potential functional importance of substitution R263K was beside confirmed by conservation analysis. Finally, we have identified in TLR4 two ligand binding positions, 339 and 386, with important amino acid substitutions that might be responsible for variability in LPS binding. No signature of positive selection was detected for these sites; however functional importance of position 386 was supported by the Consurf analysis. Intriguingly, both residues form charge interactions with the same lipid A phosphate of the LPS, which might indicate that the evolution of this position is associated with phosphate binding. However, this interpretation must be taken cautiously since Resman et al. [81] have questioned the role of the site 386 (in human K388) in LPS binding.

LBR_TLR7_ sequence was much shorter than LBR_TLR4_ one (103 vs. 222 codons, respectively), which could be explained by the smaller size of LBR_TLR7_ ligand, the viral ssRNA [68]. LBR_TLR7_ was highly conserved at the interspecific level. Only two rapidly evolving positions (out of 103 analysed sites) were detected and neither of them corresponded to the predicted ligand binding residues [68]. Generally the conserved sites (sites evolving under negative selection), have important evolutionary roles for example in protein-protein interactions (TIR domain) or in the preservation of protein structure (*e*.*g*. LRR forming horseshoe structure).

We found that structural variation between rodent LBR of both TLRs (TLR4 - 1.5Å and TLR7 - 1.7Å) was comparable with the variation observed between ECD_TLR4_ of human and mouse (1.7Å). The 3D-protein structure modeling revealed that LBR_TLR4_ differed between *Rattus sakeratensis*, *R*. *nitidus* and all other rodent species. The analysis of LBR_TLR4_ sequences did not reveal any specific or unique substitution that could be responsible for this clustering. The same analysis performed on LBR_TLR7_ revealed that *Rattus exulans* substantially differed from other species. This difference could be explained by substitutions found at position H516Y, one being specific of *R*. *exulans* (Y at position 516) while other *Rattus* and *Mus* species harbored an H amino acid at this position. These inter-specific differences in LBR 3D structure were not related to the phylogenetic distance between species. They could be better explained by similar pathogen exposition and thus similar pathogen-mediated selection.

The results of charge analyses might be more important as they revealed interspecific variation in LBRs of both receptors. *Mus* species had generally a more negative overall charge at LBR than *Rattus* species (Additional file 1: Figure S6). Differences in protein charges were previously shown to be associated with differences in protein-ligand interactions [41,65]. Likewise, differences between these two groups were also found in LBR_TLR4_ at positions that directly bind to LPS. However, some caution is needed, since variation of TLR4 and TLR7 in sensitivity to LPS or ssRNA, respectively, between rats and mice has not been investigated.

### Differences in evolution of bacterial-sensing and viral-sensing Tlrs

Our results showed that the bacterial-sensing *Tlr*4 was more variable than the viral-sensing *Tlr*7, and that *Tlr*4 evolution was more intensively shaped by positive selection than in *Tlr*7. *Tlr*4 had 1.7% of codons under positive selection, while in *Tlr*7 it was only 0.38%. These differences are likely to be explained by *Tlr*s’ specificity to different groups of MAMPs with which they co-evolved [56]. *Tlr*4 detects more types of ligands (*e*.*g*. bacterial LPS, envelope viral components, fungal cell wall components – Mannan) [30] and it seems that these pathogen structures have exerted more diversifying selective pressures on *Tlr*4 than the viral ssRNA affecting *Tlr*7. Recent studies of parasites show that there is an important structural variability in MAMPs between bacterial species (*e*.*g*. flagellin and LPS) [44,81,83-87]. We propose that the ligand binding region of *Tlr*4 detecting these MAMPs should reflect higher ligand variability observed in our data.

Reduced genetic variability in important genes generally results from strong purifying selection acting against deleterious mutations in these genes [88]. It can result in a smaller effective population size and a lower amount of incomplete lineage sorting [72,89]. These two phenomena were found to be more pronounced when analysing *Tlr*7 phylogeny. Moreover the *Tlr*7 gene is located on the X chromosome in mammals, which can be advantageous during evolution (*e*.*g*. lower polymorphism is maintained by quicker fixation of beneficial mutations and elimination of deleterious ones by stronger selection and more intense genetic drift) [90]. We suggest that the tension between diversifying and purifying selection, caused by adaptation to the variability of viral motifs detected by viral-sensing *Tlr*7 and maintenance of function together played an important role in the distribution of *Tlr*7 polymorphisms.

## Conclusion

This study brings a unique insight into the natural variability and molecular history of two Toll-like receptors in free-living populations of 23 murine species. Purifying selection seems to be the dominant evolutionary force shaping *Tlr*4 and *Tlr*7 polymorphism. However, specific sites putatively evolving under diversifying selection were detected in both *Tlr*s. These sites accumulated within *Tlr*4 LBR, and detailed analyses revealed that several important amino-acid substitutions might alter LPS binding. These substitutions were often species-specific and differentiated between the Rattini and Murini tribes. Interspecific charge variability of LBR and to lesser extent the variability in 3D structure indicated the potential differences in protein-ligand interaction. By contrast, the evolution of *Tlr*7 was strongly shaped by purifying selection. All predicted ligand binding residues in this receptor were uniform across all studied mammals to date. The contrasting evolutionary histories of these two *Tlr*s are likely to result from different structural variability of ligands they target. Since the crystallography of certain ligands (*e*.*g*. biglycans, hyaluronans and heparin sulphates, ssRNA) [44,68] remains unknown and the precise positions of corresponding binding sites are still missing, our data provide important avenues towards understanding which codons might be candidates for ligand binding residues.

## Methods

### Sampling

Murine rodents from 23 species belonging to the Rattini and Murini (sensu Lecompte et al. [91]) tribes were sampled mainly in South-East Asia, and three synanthropic species (*i*.*e*. *Rattus rattus*, *Mus m*. *muscululus* and *Mus m*. *domesticus*) were also sampled in Europe and Africa. In our sampling area, *Rattus tanezumi* specimens corresponded to two divergent mitochondrial lineages although they could not be distinguished according to their nuclear pool [73]. These samples were further referred to clades *R*. *tanezumi R2* and *R3* according to their mitotype. *Rattus sakeratensis* corresponds to the lineage previously referred to as *R*. *losea* and found in central, northern Thailand and Vientiane Plain of Lao PDR (*Rattus losea*-like by Pagès et al. [69]). This lineage was recently distinguished from the true *R*. *losea*, which is restricted to Cambodia, Vietnam, China and Taiwan [70].

Species identification was initially based on morphological criteria and thereafter confirmed using molecular barcoding for problematic lineages [69,92]. We sequenced two to 10 individuals per species. In total 103 specimens were analysed (Additional file 1: Table S1).

### Toll-like receptor sequencing and sequence alignments

We sequenced the complete exon 3 of *Tlr*4 (2.250 bp) and *Tlr*7 (3.150 bp) as it encompasses the LBR in both genes. Exon 3 corresponds to 89.7% and 99.0% of the total coding sequence for *Tlr*4 and *Tlr*7, respectively. Short exons 1 and 2 (241 bp encoding 5´- untranslated (UT) region and first 257 bp of ECD in *Tlr*4_exon2_ and 154 bp of 5´-UT regions and 3bp of ECD in *Tlr*7_exon2_) were not analysed in present study, because we were preferentially interested by functional regions (*e*.*g*. LBR and TIR). For all analyses and discussion the codon numbering follows the sequences of *Rattus norvegicus* available in GenBank [GenBank Acc. NP_062051.1 for *Tlr*4, and NP_001091051.1, for *Tlr*7].

Primers for Polymerase Chain Reaction (PCR) and sequencing were designed according to the sequences available in the Ensembl database for *Mus musculus* [*Tlr*4 ENSMUSE00000354724/MGI:96824, *Tlr*7 ENSMUSE00000405820/ MGI:2176882] and *Rattus norvegicus* [*Tlr*4 ENSRNOE00000099045/NP_062051, *Tlr*7 ENSRNOE00000039897/NP_001091051]. We used the software Primer3[93] to design primers (see their sequences in Additional file 1: Table S2 and positions in Additional file 1: Figure S1). Total DNA was extracted from rodent tissue (biopsy from ear or necropsy from liver) using the DNeasy Blood & Tissue Kit (Qiagen AB, Hilden, Germany). Amplifications were carried out in a final volume of 25 μl containing 12.5 μl of Multiplex Kit PCR master mix (Qiagen), 9.3 μl of H_2_O, 0.5 μM of each of primer pairs and 2 μl of DNA. Cycling conditions included an initial denaturation at 95°C for 15 min, followed by 10 cycles of denaturation at 95°C for 40 s, annealing with touchdown at 65°C to 55°C (-1°C/cycle) for 45 s and extension at 72°C for 90 s, followed by 30 cycles of denaturation at 95°C for 40 s, annealing at 55°C for 45 s and extension at 72°C for 90 s, with a final extension phase at 72°C for 10 min. The final extension was performed for 10 min at 72°C. The lengths of amplicons were checked on 1.5% agarose gels. Sequencing was carried out using an ABI3130 automated DNA sequencer (Applied Biosystems). DNA sequences were aligned and edited using SeqScape v.2.5 (Applied Biosystems) and BioEdit v.7.1.3 (Hall 1999). All sequences have been submitted to NCBI GenBank (Accession numbers are presented in Additional file 1: Table S1).

### Sequence analysis

Diploid genotypes were resolved using the Bayesian PHASE platform [94] implemented in DnaSP ver. 5.10 [95]. Calculations were carried out using 1000 iterations, 10 thinning intervals, and 1000 burn-in iterations. Sequences were collapsed into individual alleles by Fabox DNA collapser, an online FASTA sequence toolbox [96]. The identification and visualization of main domains (ECD, TM and ICD with TIR domain and ICD-DP) was performed in SMART [97] based on *Rattus norvegicus* sequences provided in GenBank [NP_062051.1 for *Tlr*4 and NP_001091051.1 for *Tlr*7]. 3D structure was predicted in Phyre2 [98] and then visualized using FirstGlance in Jmol v.1.9. Finally, we estimated nucleotide diversity (π), number of polymorphic sites (*S*) and total number of mutations (ϵ) with DnaSP, and the number of nucleotide alleles (*hN*) and amino acid variants (*hA*) using Fabox DNA collapser.

### Phylogenetic reconstructions and congruence between the tree based on a comparably sized non-immune sequence dataset and Tlr trees

We first tested *Tlr* sequences for recombination using SBP, to avoid further false positive events of selection. This method (implemented in Datamonkey,[66,99]) allowed the screening of *Tlr* sequences for recombination breakpoints. SBP identify non-recombinant regions and allowed each region to have its own phylogenetic reconstruction [100,101].

Phylogenies were reconstructed independently for each gene using the alignment of complete exon 3 sequences. A phylogeny inferred from the combination of one nuclear (the first exon of the gene encoding the interphotoreceptor retinoid binding protein, *Irbp*) and two mitochondrial genes (the cytochrome *b* gene, *Cytb*, and the cytochrome *c* oxidase I gene, *Co*I), taken from Pagès et al. [69], was used for comparison of “neutral” evolution of the studied rodents with trees obtained from the immune gene alignments. Both Maximum likelihood (ML) and Bayesian (BA) methods were applied to infer phylogenetic relationships from each *Tlr* alignments. The best evolutionary model of nucleotide substitution was determined using jModelTest 0.1.1 [102]. Phylogenies based on ML analyses were reconstructed using RAxML 7.2.6 [103]. Analyses were run as the rapid bootstrap procedure (option –f a) with bootstraps defined by option –NautoMR. For both *Tlr*s we used nucleotide substitution model GTR + Γ (option –m GTRGAMMA) selected by jModelTest 0.1.1 as the most appropriate to our data. Bayesian analyses were performed using a parallel version of MrBayes v3.1 [104] at the University of Oslo Bioportal [105] and CBGP HPC computational platform located at Centre de Biologie et Gestion des Populations, Montpellier. Two runs of 50,000,000 generations in each were adopted, applying the best fitted model of substitution (GTR+ Γ). A burn-in period of 10,000,000 generations was determined using Tracer 1.4 [106]. Convergence was also evaluated using Tracer v1.4. After discarding samples from the burnin period, results were based on the pooled samples from the stationary phases of the two independent runs. Trees were edited using FigTree v1.3.1. [107].

We tested the congruence between the rodent phylogeny and the *Tlr*s phylogeny based on the MrBayes approach using reconciliation analyses. Reconciliation analyses explore all possible mappings of one tree onto another, assigning different costs to evolutionary events and find optimal (*i*.*e*. yielding minimal costs) solutions. These analyses were conducted using JANE 4 [108]. This software was initially built to reconcile parasite and host trees, yet it can also be used for comparative analysis of species and gene trees. In the context of host-parasite relationships, five evolutionary events between parasites and host can be taken into account in JANE 4: co-speciation, host switches, duplication, failure to diverge and parasite loss. These events are analogous to co-divergence, convergence, duplication, purifying selection and gene loss (respectively) when considered in the context of species and gene tree reconciliation. For each of these events the specific costs can be set. The lowest cost is attributed to the event considered as most likely. In order to obtain reconciliations that maximize the number of co-divergences we set the cost of a co-divergence event to 0 while other costs were set to 1 (see Cruaud et al. [109] for similar approach). The cost of the best solution is then compared with costs found in reconciliations in which tip mappings are permuted at random. This generates a null distribution of the costs of reconciliation. If the cost of the best solution is lower than that expected by the chance it means that the two phylogenies are significantly congruent. The following parameters were used: the number of generations (iterations of the algorithm) was set to 100 and the “population” (number of samples per generation) was set to 100. Input phylogenies were those obtained by the Bayesian inference. The cost of the best solution was compared to distribution of the costs of 1000 randomizations.

Moreover, we tested the congruence between genes and tree based on a comparably sized non-immune sequence dataset using SH test [110] as implemented in PAUP. Alternative topologies required for ML SH test were reconstructed by ML approach in the software Garli v. 2.0 [88]. Two different ML trees were estimated for each *Tlr*; a first one inferred under non-constrained conditions with default options and a second one constrained by the tree topology based on a comparably sized non-immune sequence dataset. Mouse species (genus *Mus*) were excluded from the analysis of co-divergence in order to match data with the study of Pagès et al. [69] where the mice are missing.

### Search for signatures of selection on Tlr sequences

We estimated separately the number of synonymous (*dS*) and non-synonymous (*dN*) substitutions per site for the whole exon 3, ECD, LBR and the TIR domains, and for both *Tlr*s. Computations were made with 1000 bootstraps and Nei-Gojobori method (with Jukes-Cantor correction) in MEGA 5 [111]. We then estimated the overall ratio *dN*/*dS* for each domain and for the whole exon 3 of both *Tlr*s by Single Likelihood Ancestor Counting (SLAC) implemented in Datamonkey. The p-value was 0.05. As the SLAC method tends to be a very conservative test, the actual rate of false positives (*i*.*e*. neutrally evolving sites incorrectly classified as selected) can be much lower than the significance level [67]. In the next step we estimated selection at each codon by SLAC to find which codons of the exons 3 have been subject to positive and negative selection. As a default tree we used a NJ tree and appropriate substitution model proposed by automatic model selection tool in Datamonkey.

Finally, we used the Mixed Effects Model of Evolution (MEME) algorithm in the Hyphy package accessed on the website of Datamonkey interface [99] to detect codons evolved under positive selection along the branches of the phylogenies. This method is recently recommended as a replacement for the Fixed Effects Likelihood (FEL) and SLAC models [67]. It allows the detection of signatures of episodic selection, even when the majority of lineages are subject to purifying selection. This test permits ω to vary from site to site and also from branch to branch in phylogeny [67]. Tests of episodic diversifying selection were performed at significance level p < 0.05 and MrBayes trees were used as working topologies. Only events of positive selection with Empirical Bayes Factor (EBF) estimated by MEME near to 100 were mapped on to the phylogeny.

### Functional analysis of ligand binding region

Positions of LBR in both TLRs have been previously described in humans [39,68]. The corresponding LBR position in rodents was predicted based on the human-rodent alignment. The LBR was located between codons AA248 and AA469 in TLR4 and between codons AA495 and AA597 in TLR7.

We first explored the evolutionary conservation of each amino acid position in LBR using the Consurf algorithm [112]. Consurf estimates the evolutionary rate of amino acid positions in a protein molecule, based on the phylogenetic relationships between homologous sequences. Conservation scale is defined from the most variable amino acid positions (grade 1, color represented by turquoise) which are considered as rapidly evolving to conservative positions (grade 9, color represented by maroon) which are considered as slowly evolving. We used the proposed substitution matrix and computation was based on the empirical Bayesian paradigm. MrBayes trees were used as the working topology. Protein tertiary structure was adopted from *R*. *norvegicus* [Gene Bank Acc. TLR4/KC811688 and TLR7/KC811786].

Because protein tertiary structure is essential for its biological function we finally explored the variability in the 3D structures of LBRs in the different AA variants. The prediction of 3D structures of the variants was performed by homology modeling using Phyre2 [98]. Differences in 3D protein structure among variants were then evaluated using the root mean square deviations (RMSD) calculated by the DALI pairwise comparison tool [113]. The RMSD-based distance matrices were analysed in STATISTICA v. 8.0 (StatSoft, Inc., Tulsa) by joining tree clustering using Unweighted Pair Group Method with Arithmetic Mean (UPGMA, [114]). We then analysed the variability of the charge of each LBR variant, which could be another key indicator of functional changes, because differences in protein charge could influence the ability to bind ligands [41,65]. LBR charge of each variant was estimated at predefined neutral pH = 7 using LRRfinder[115].

## Availability of supporting data section

All sequences have been submitted to NCBI GenBank under Accession numbers from KC811609 to KC811800 (Individual accession numbers are presented in Additional file 1: Table S1). *Tlr* phylogenies based on MrBayes (Tlr4_MrBayes_final.nex, Tlr7_MrBayes_final.nex) and RAxML (Tlr4_RAxML_final.nex, Tlr7_RAxML_final.nex) approach were added to the TreeBase database (http://treebase.org/treebase-web/home.html). Trees are available at URL: http://purl.org/phylo/treebase/phylows/study/TB2:S14659.

## Competing interests

The authors declare that they have no competing interests.

## Authors’ contributions

Conceived and designed the experiments: AF JFC JB NCH MV. Performed the sequencing: AF MG FC. Analysed the data: AF MV MP EJ. Contributed samples: SM JFC AF. Wrote the paper: AF MV JFC JB NCH MP EJ (sorted by the significance of contributions). All authors read and approved the final manuscript.

## Supplementary Material

Additional file 1: Table S1 Summary of sampled specimens and identification of haplotypes. **Table S2.** Primer description. **Table S3.** Residues binding to LPS in TLR4 based on knowledge of 3D-crystalography in human predicted by Park et al. 2009. **Table S4.** Potential residues binding ssRNA predicted by Wei et al. 2009. **Figure S1.** Protein structure of TLR4 (a, c) and TLR7 (b, d) identified by SMART (http://smart.embl-heidelberg.de/) (a, b) and CONSURF (c, d). SMART (a, b) identified following types of domains: **LRR** - Leucine rich repeat; **LRRCT** - Leucine rich repeat C-terminal domain; **TIR** - TIR domain, **Fulfilled blue box (TD)** - transmembrane domain; **LRRNT** - Leucine rich repeat N-terminal domain. **Red box** - LBR (from AA248 to AA469 for TLR4 and from AA495 to AA597 for TLR7). **ECD** - extracellular domain is represented by solid black double arrow; **ICD** - intracellular domain is represented by dashed double arrow. Distal part of ICD **(ICD-DP)** is indicated by a simple solid arrow. Positions of forward and reverse primers used for amplification are shown by arrows. Arrows of same color indicates primer pairs. Description of crystallographic structure (c, d) **LBR** is represented by red polygon; **TD** is present between two dashed lines. To the right from TD is **ICD**, to the left is **ECD**. **Figures S4.** Test of congruence between the presumably neutral and *Tlr* phylogenies (*Tlr*4 (a), *Tlr*7 (b) following JANE 4). Number at X axis represents costs of co-divergence. The red dashed line represents the cost observed in our data. The blue columns represent the random distributions of costs. Lower cost than random observed in our data signified higher congruence between species and gene topologies. **Figure S5.** Superimposition of structures, tree clustering diagrams based on linkage distance, (a) LBRTLR4 and (b) LBRTLR7; individual LBR-variants often unify more species; description of LBR-variants labels is in the Table S1 under Hap_LBRTLR4 and Hap_LBRTLR7. **Figure S6.** Analysis of LBR amino acid sequence charge at pH 7 (LRRFinder) for (a) LBRTLR4 and (b) LBRTLR7, individual LBR-variants often unify more species; description of LBR-variants labels is in the Table S1 under Hap_LBRTLR4 and Hap_LBRTLR7. Mouse species are in red, *Rattus* spp. and related genera are in blue. Click here for file

Additional file 2: Figures S2 and S3 (Phylogenetic trees). Click here for file
