# Supplementary material for: Contrasted evolutionary histories of two Toll-like receptors (Tlr4 and Tlr7) in wild rodents (MURINAE)
Source: BMC Evol Biol. 2013 Sep 12;13:194. doi: 10.1186/1471-2148-13-194 (PMC3848458; doi:10.1186/1471-2148-13-194)

ADDITIONAL FILES 1

TABLES

Table S1. Summary of sampled specimens and identification of haplotypes.

ID	Species	Country	Province	Sex	LBR$_{TLR4}$ variants	LBR$_{TLR7}$ variants	Hap Exon 3 *Tlr4*	Hap Exon 3 *Tlr7*	GenBank Acc. *Tlr4*/*Tlr7*
**C0443**	*Bandicota indica*	Cambodia	Mondolkiri	F	Bain1_Bain2	Rattus sp.	**bainh5_bainh6**	**bainh1**	**KC811609/KC811705**
**L0276**	*Bandicota indica*	Lao PDR	Champasak	M	Bain3	Rattus sp.	**bainh1_bainh2**	**bainh2**	**KC811610/KC811706**
**R4000**	*Bandicota indica*	Thailand	Kalasin	M	Bain1	Rattus sp.	**bainh4**	**bainh3**	**KC811611/KC811707**
**R5313**	*Bandicota indica*	Thailand	Nan	M	Bain3	Rattus sp.	**bainh3**	**bainh4**	**KC811612/KC811708**
**C0709**	*Bandicota savilei*	Cambodia	Mondolkiri	F	Basa2	Basa	**basah1**	basah2	**KC811613/KC811709**
**L0331**	*Bandicota savilei*	Lao PDR	Champasak	F	Basa1_Basa2	Basa	**basah2_basah3**	**basah1**_basah2	**KC811614/KC811710**
**R4141**	*Bandicota savilei*	Thailand	Phrae	M	Basa3	Basa	**basah4**	**basah2**	**KC811615/ KC811711**
**R5475**	*Bandicota savilei*	Thailand	Buriram	F	Basa1	Basa	**basah5_basah6**	basah2	**KC811616/ KC811712**
**C0333**	*Berylmys berdmorei*	Cambodia	Sihanouk	M	Bebe	Be sp.	**bebeh2**	**bebeh1**	**KC811617/ KC811713**
**C0481**	*Berylmys berdmorei*	Cambodia	Mondolkiri	M	Bebe	Be sp.	**bebeh1**	**bebeh2**	**KC811618/ KC811714**
**L0006**	*Berylmys berdmorei*	Lao PDR	Luang Prabang	M	Bebe	Be sp.	bebeh3_**bebeh4**	**bebeh3**	**KC811619/ KC811715**
**R3441**	*Berylmys berdmorei*	Thailand	Loei	M	Bebe	Be sp.	**bebeh3**	**bebeh4**	**KC811620/ KC811716**
**L0151**	*Berylmys bowersi*	Lao PDR	Luang Prabang	F	Bebo	Be sp.	**beboh4**_beboh6	**beboh1**_beboh4	**KC811621/ KC811717**
**R4400**	*Berylmys bowersi*	Thailand	Loei	M	Bebo	Be sp.	**beboh1**	beboh4	**KC811622/ KC811718**
**R4804**	*Berylmys bowersi*	Thailand	Loei	M	Bebo	Be sp.	**beboh2_beboh3**	**beboh4**	**KC811623/ KC811719**
**R5410**	*Berylmys bowersi*	Thailand	Nan	F	Bebo	Be sp.	**beboh5_beboh6**	**beboh2_beboh3**	**KC811624/ KC811720**
**C0421**	*Leopoldamys edwardsi*	Cambodia	Mondolkiri	M	Leed	Le sp.	**leedh4_leedh5**	**leedh3**	**KC811625/ KC811721**
**R4070**	*Leopoldamys edwardsi*	Thailand	Loei	M	Leed	Le sp.	**leedh6_leedh7**	**leedh1**	**KC811626/ KC811722**
**R4276**	*Leopoldamys edwardsi*	Thailand	Phrae	F	Leed	Le sp.	**leedh1_leedh3**	leedh2	**KC811627/ KC811723**
**R4296**	*Leopoldamys edwardsi*	Thailand	Phrae	F	Leed	Le sp.	leedh1_**leedh2**	leedh2	**KC811628/ KC811724**
**R4350**	*Leopoldamys edwardsi*	Thailand	Phrae	?	x	Le sp.	x	leedh2	x/ **KC811725**
**R5057**	*Leopoldamys edwardsi*	Thailand	Loei	M	x	Le sp.	x	**leedh2**	x/ **KC811726**
**R4477**	*Leopoldamys neilli*	Thailand	Phrae	F	Lene	Le sp.	**leneh2**_leneh4	**leneh1**	**KC811629/ KC811727**
**R4486**	*Leopoldamys neilli*	Thailand	Phrae	F	Lene	Le sp.	**leneh1**_leneh4	leneh1	**KC811630/ KC811728**
**R4527**	*Leopoldamys neilli*	Thailand	Loei	F	Lene	Le sp.	**leneh4**	leneh2	**KC811631/ KC811729**
**R4530**	*Leopoldamys neilli*	Thailand	Loei	M	Lene	Le sp.	**leneh3**_leneh4	**leneh2**	**KC811632/ KC811730**
**R3033**	*Leopoldamys sabanus*	Thailand	Kanchanaburi	F	Lesa	Le sp.	**lesah1**_lesah2	**lesah1**_lesah2	**KC811633/ KC811731**
**R3111**	*Leopoldamys sabanus*	Thailand	Kanchanaburi	M	Lesa	Le sp.	lesah1_**lesah2**	**lesah2**	**KC811634/ KC811732**
**C0118**	*Maxomys surifer*	Cambodia	Sihanouk	M	Masu1	x	**masuh5_masuh7**	x	**KC811635/** x
**C0478**	*Maxomys surifer*	Cambodia	Mondolkiri	M	Masu2	x	**masuh1_masuh2**	x	**KC811636/** x
**L0274**	*Maxomys surifer*	Lao PDR	Champasak	F	Masu1	x	**masuh6**	x	**KC811637/** x
**R4099**	*Maxomys surifer*	Thailand	Loei	M	Masu3_Masu4	x	**masuh3_masuh4**	x	**KC811638/**x
**C0423**	*Mus caroli*	Cambodia	Mondolkiri	M	Muca2	Mus sp.	**mucah3**	**mucah4**	**KC811639/ KC811733**
**L0014**	*Mus caroli*	Lao PDR	Luang Prabang	F	Muca1	Mus sp.	**mucah2**	**mucah1**	**KC811640/ KC811734**
**L0211**	*Mus caroli*	Lao PDR	Luang Prabang	M	Muca1	Mus sp.	mucah3_**mucah4**	mucah1	**KC811641/ KC811735**
**L0275**	*Mus caroli*	Lao PDR	Champasak	M	Muca3	Mus sp.	**mucah1**_mucah3	**mucah2**	**KC811642/ KC811736**
**R5642**	*Mus caroli*	Thailand	Buriram	M	x	Mus sp.	x	**mucah3**	x/ **KC811737**
**R4864**	*Mus cervicolor*	Thailand	Loei	F	Muce1_Muce2	Mus sp.	**muceh4_muceh5**	**muceh1**	**KC811643/ KC811738**
**R5644**	*Mus cervicolor*	Thailand	Buriram	M	Muce1_Muce2	Mus sp.	**muceh1_muceh2**	**muceh2**	**KC811644/ KC811739**
**R5666**	*Mus cervicolor*	Thailand	Buriram	F	x	Mus sp.	x	muceh2	x/ **KC811740**
**R5671**	*Mus cervicolor*	Thailand	Buriram	F	Muce2	Mus sp.	**muceh3**_muceh2	**muceh3**	**KC811645/ KC811741**
**L0103**	*Mus cookii*	Lao PDR	Luang Prabang	F	Muco1	Mus sp.	mucoh3	**mucoh3**	**KC811646/ KC811742**
**L0178**	*Mus cookii*	Lao PDR	Luang Prabang	M	Muco1	Mus sp.	**mucoh3**	mucoh3	**KC811647/ KC811743**
**R4106**	*Mus cookii*	Thailand	Loei	F	Muco1_Muco2	Mus sp.	**mucoh1_mucoh2**	**mucoh1_mucoh2**	**KC811648/ KC811744**

**SK1515**	*Mus domesticus*	France	Saint Jean-et-Royans	F	Mus sp.	Mus sp.	**mudoh5**	**mudoh3**	**KC811649/ KC811745**
**SU3770**	*Mus domesticus*	Turkey	Izmir	F	Mus sp.	Mus sp.	**mudoh2**_mudoh5	mudoh1_mudoh3	**KC811650/ KC811746**
**M273**	*Mus domesticus*	Syria	Palmyra	M	Mus sp.	Mus sp.	**mudoh4**_mudoh5	**mudoh1**	**KC811651/ KC811747**
**MISC352**	*Mus domesticus*	Egypt	Sabha Oasis	M	Mus sp.	Mus sp.	**mudoh1_mudoh3**	**mudoh2**	**KC811652/ KC811748**
**JPC2821**	*Mus musculus*	Czech rep.	Buskovice	F	Mus sp.	Mus sp.	**mumuh3**	mumuh2	**KC811653/ KC811749**
**SK843**	*Mus musculus*	Germany	Lindhorst	M	Mumu2	Mus sp.	mumuh4	mumuh2	**KC811654/ KC811750**
**SU5218**	*Mus musculus*	Russia	Shkili	F	Mumu2	Mus sp.	mumuh4	mumuh1_mumuh2	**KC811655/ KC811751**
**360**	*Mus musculus*	Romania	Botosani	M	Mumu2	Mus sp.	mumuh3_**mumuh4**	**mumuh2**	**KC811656/ KC811752**
**SU5209**	*Mus musculus*	Ukraine	Primorskoe	M	Mus sp._Mumu1	Mus sp.	**mumuh1_mumuh2**	**mumuh1**	**KC811657/ KC811753**
**C0322**	*Niviventer fulvescens*	Cambodia	Sihanouk	F	Nifu1	Nifu	**nifuh1_nifuh2**	**nifuh2**	**KC811658/ KC811754**
**C0430**	*Niviventer fulvescens*	Cambodia	Mondolkiri	F	x	Nifu	x	**nifuh5_nifuh8**	x/ **KC811755**
**L0273**	*Niviventer fulvescens*	Lao PDR	Champasak	F	Nifu1	Nifu	**nifuh3_nifuh4**	**nifuh6_nifuh7**	**KC811659/ KC811756**
**R4071**	*Niviventer fulvescens*	Thailand	Loei	M	Nifu1_Nufu2	Nifu	**nifuh5_nifuh6**	**nifuh1**	**KC811660/ KC811757**
**R4497**	*Niviventer fulvescens*	Thailand	Phrae	F	Nifu1	Nifu	nifuh3_**nifuh7**	**nifuh3_nifuh4**	**KC811661/ KC811758**
**L0052**	*Rattus andamanensis*	Lao PDR	Luang Prabang	F	Raan1	Rattus sp.	**raanh1**	raanh1_raanh2	**KC811662/ KC811759**
**L0149**	*Rattus andamanensis*	Lao PDR	Luang Prabang	F	Raan2_Raan3	Rattus sp.	**raanh2_raanh3**	**raanh1_raanh2**	**KC811663/ KC811760**
**R2953**	*Rattus andamanensis*	Thailand	Kanchanaburi	F	Raan3	Rattus sp.	raanh4_**raanh5**	raanh3_**raanh4**	**KC811664/ KC811761**
**R3087**	*Rattus andamanensis*	Thailand	Kanchanaburi	M	Raan3	Rattus sp.	**raanh4**_raanh5	**raanh3**	**KC811665/ KC811762**
**C0014**	*Rattus argentiventer*	Cambodia	Sihanouk	M	Raan1	Rattus sp.	**raarh5**	**raarh1**	**KC811666/ KC811763**
**C0048**	*Rattus argentiventer*	Cambodia	Sihanouk	F	Raan1	Rattus sp.	**raarh3**_raanr5	raarh1_**raarh2**	**KC811667/ KC811764**
**C0104**	*Rattus argentiventer*	Cambodia	Sihanouk	M	Raan1	Rattus sp.	**raarh2**	raarh1	**KC811668/ KC811765**
**R5674**	*Rattus argentiventer*	Thailand	Buriram	F	Raan1	Rattus sp.	**raarh1**	raarh1	**KC811669/ KC811766**
**R5679**	*Rattus argentiventer*	Thailand	Buriram	M	Raar	Rattus sp.	**raarh4**	raarh1	**KC811670/ KC811767**
**C0278**	*Rattus exulans*	Cambodia	Sihanouk	M	Raex1	Raex	**raexh1_raexh3**	**raexh5**	**KC811671/ KC811768**
**C0353**	*Rattus exulans*	Cambodia	Mondolkiri	F	Raex3	Raex	raexh1_**raexh2**	**raexh2**_raexh5	**KC811672/ KC811769**
**L0217**	*Rattus exulans*	Lao PDR	Champasak	F	Raex1	Raex	**raexh7**	**raexh4**_raexh5	**KC811673/ KC811770**
**R1805**	*Rattus exulans*	Thailand	Bangkok	M	Raex1	Raex	**raexh5_raexh6**	**raexh3**	**KC811674/ KC811771**
**R4103**	*Rattus exulans*	Thailand	Loei	M	Raex2	Raex	**raexh4**_raexh7	**raexh1**	**KC811675/ KC811772**
**L0277**	*Rattus sakeratensis*	Lao PDR	Champasak	F	Rasa	Rattus sp.	rasah5_**rasah6**	**rasah2**	**KC811676/ KC811773**
**R0237**	*Rattus sakeratensis*	Thailand	Ratchaburi	F	Rasa	Rattus sp.	**rasah1**_rasah6	**rasah1**_rasah5	**KC811677/ KC811774**
**R1015**	*Rattus sakeratensis*	Thailand	Nakhon Ratchasima	M	Rasa	Rattus sp.	**rasah3_rasah5**	**rasah3**	**KC811678/ KC811775**
**R4402**	*Rattus sakeratensis*	Thailand	Loei	F	Rasa	Rattus sp.	**rasah4**_rasah5	**rasah5**	**KC811679/ KC811776**
**R4568**	*Rattus sakeratensis*	Thailand	Phrae	M	Rasa	Rattus sp.	**rasah2**	**rasah4**	**KC811680/ KC811777**
**L0180**	*Rattus nitidus*	Lao PDR	Luang Prabang	F	Rani	Rano-Rani	**ranih5**	**ranih1**	**KC811681/ KC811778**
**L0191**	*Rattus nitidus*	Lao PDR	Luang Prabang	M	Rani	Rano-Rani	**ranih3**_ranih5	ranih1	**KC811682/ KC811779**
**L0192**	*Rattus nitidus*	Lao PDR	Luang Prabang	F	Rani	Rano-Rani	ranih1_**ranih4**	ranih1	**KC811683/ KC811780**
**L0196**	*Rattus nitidus*	Lao PDR	Luang Prabang	M	x	Rano-Rani	x	ranih1	x/ **KC811781**
**R4846**	*Rattus nitidus*	Thailand	Loei	M	Rani	Rano-Rani	**ranih1_ranih2**	ranih1	**KC811684/ KC811782**
**C0141**	*Rattus norvegicus*	Cambodia	Sihanouk	F	Rano	Rano-Rani	ranoh1	**ranoh1**_ranoh3	**KC811685/ KC811783**
**C0210**	*Rattus norvegicus*	Cambodia	Sihanouk	F	Rano	Rano-Rani	ranoh1	**ranoh2**_ranoh3	**KC811686/ KC811784**
**C0211**	*Rattus norvegicus*	Cambodia	Sihanouk	F	Rano	Rano-Rani	ranoh1	**ranoh3**	**KC811687/ KC811785**
**C0224**	*Rattus norvegicus*	Cambodia	Sihanouk	F	Rano	Rano-Rani	**ranoh1**	ranoh3	**KC811688/ KC811786**
**C0028**	*Rattus tanezumi R3*	Cambodia	Sihanouk	F	Rata7_Rata8	Rattus sp.	**ratah1_ratah7**	**ratah7_ratah8**	**KC811689/ KC811787**
**C0250**	*Rattus tanezumi R3*	Cambodia	Sihanouk	F	Rati_Rata9	Rattus sp.	**ratah4_ratah5**	**ratah1**	**KC811690/ KC811788**
**C0477**	*Rattus tanezumi R3*	Cambodia	Mondolkiri	M	Rata9	Rattus sp.	**ratah6**	**ratah4**	**KC811691/ KC811789**
**L0313**	*Rattus tanezumi R3*	Lao PDR	Champasak	F	x	Rattus sp.	x	**ratah11_ratah12**	x/ **KC811790**
**L0242**	*Rattus tanezumi R3*	Lao PDR	Champasak	F	Rata9	Rattus sp.	ratah6_**ratah8**	**ratah5_ratah6**	**KC811692/ KC811791**
**R5051**	*Rattus tanezumi R3*	Thailand	Loei	F	Rata10_Rata11	Rattus sp.	**ratah2_ratah3**	**ratah2_ratah3**	**KC811693/ KC811792**
**NK37**	*Rattus rattus*	Senegal	?	F	Rara	Rattus sp.	rarah1	rarah1	**KC811694/ KC811793**
**R197**	*Rattus rattus*	Guadaloupe	?	?	Rara	Rattus sp.	**rarah1**	**rarah1**	**KC811695/ KC811794**
**R2**	*Rattus rattus*	Riov	?	?	Rara	Rattus sp.	rarah1	rarah1	**KC811696/ KC811795**
**L0100**	*Rattus tanezumi R2*	Lao PDR	Luang Prabang	F	Rata1_Rata2	Rattus sp.	**ratah10_ratah11**	ratah9_**ratah10**	**KC811697/ KC811796**

**R1831**	*Rattus tanezumi R2*	Thailand	Nakhon Sri Thammarat	F	Rata3_Rata4	Rattus sp.	**ratah15_ratah16**	**ratah9**	**KC811698/ KC811797**
**R3560**	*Rattus tanezumi R2*	Thailand	Samui	F	Rati_Rata5	Rattus sp.	**ratah13_ratah14**	**ratah13**	**KC811699/ KC811798**
**R4377**	*Rattus tanezumi R2*	Thailand	Loei	M	Rata1_Rata6	Rattus sp.	**ratah9_ratah12**	ratah9	**KC811700/ KC811799**
**Li0249**	*Rattus tiomanicus*	Sumatra	Minas	M	Rati	x	**ratih1_ratih2**	x	**KC811701/** x
**Li0258**	*Rattus tiomanicus*	Sumatra	Minas	M	Rati	x	**ratih3**_ratih6	x	**KC811702/** x
**Li0259**	*Rattus tiomanicus*	Sumatra	Minas	M	Rati	x	**ratih4**_ratih6	x	**KC811703/** x
**Li0315**	*Rattus tiomanicus*	Sumatra	Minas	?	Rati	Rattus sp.	**ratih5_ratih6**	**ratih1**	**KC811704/ KC811800**

NOTE. - ID - identification of specimens; **LBR_{TLR4} and LBR_{TLR7} variants** - variants of ligand-binding region, double indication mean heterozygote specimen; **Hap_Exon3_*Tlr*4** and ***Tlr*7**- alleles of exon 3 for each species, double indication means heterozygote specimen; haplotypes used for all analysis are in bold; **x** - sequence not complete;**? -** no exact information; **GenBank Acc.** - Gen Bank Accession numbers.

Table S2. Primer description.

Primer ID	Sequence of primers 5' – 3'	Function
rTLR4-F	AGT TTA TCA TCA CTG YA GCA AG	amplification
rTLR4-XF2	CCC AAT TGA CTC CAT TCA AGC CC	amplification
rTLR4-XF3	CCC TCA GGA CTC TTG ATT GCA G	amplification
rTLR4-R-1	ATT CTC CCA AGA TCA ACC GAT G	amplification
rTLR4-R-3	CTG KTC CTT GAC CCA CTG C	amplification
rTLR4-R	AGA RMC CCA GRT GAR CTG TAG CAT T	amplification
rTLR7-F	AAG ACC YRT GTT GYT TAG TTT TAA TAA TG	amplification
rTLR7-1F	CAG ATT AGA CCT GGA AGC TTT AGT G	amplification
rTLR7-4F	TCT TGA CCT TGG CAC TAA CTT CAT A	amplification
rTLR7-5F	CCA TTG GCC AAA CTC TTA ATG G	sequencing
rTLR7-6F	GGT GAT AAC AGA TAC TTG GAC TTC T	sequencing
rTLR7-7F	CTG GCC ACT GAT GTG ACT TGT	sequencing
rTLR7-2R	GTT AGC CTC AAG GCT CAG AAG	amplification
rTLR7-9R	TAT CGG AAA TAG TGT AAG GCC TCA AG	amplification
rTLR7-R	AGA AAG AAR TTA TCK TCT ATC AGT CTC	amplification

Table S3. Residues binding to LPS in TLR4 based on knowledge of 3D-crystalography in human predicted by Park et al. 2009.

Position in rodent alignment	Numbering in human sequence	Function of AA	Residue variety in rodents
<u>263</u>	hTLR4_R264	LPS (Charge interaction with phosphates)	positively charged R, K
339	hTLR4_K341	LPS (Charge interaction with phosphates)	positively charged R, H, uncharged NH2 residue Q
360	hTLR4_K362	LPS (Charge interaction with phosphates)	positively charged K, R
<u>386</u>	hTLR4_K388	LPS (Charge interaction with phosphates)	positively charged R, uncharged hydrophilic T, small uncharged hydrophilic S, uncharged hydrophobic I
434	hTLR4_Q436	LPS (Hydrogen bond)	uncharged R, K
438	hTLR4_F440	LPS, MD-2 (Hydrophobic interaction)	uniformly aromotic hydrophobic F
442*	hTLR4_L444	LPS, MD-2 (Hydrophobic interaction)	aliphatic hydrophobic L, aromatic polar hydrophobic Y
461	hTLR4_F463	LPS, MD-2 (Hydrophobic interaction)	uniformly aromotic hydrophobic F

NOTE. - Variable sites detected by CONSURF are underlined; * indicates sites identified by MEME.

Table S4. Potential residues binding ssRNA predicted by Wei et al. 2009.

Position in rodent alignment	Numbering in human sequence	Function of AA	Residue variety in rodents
503	hTLR7_K502	Potential ligand binding residue	uniformly positively charged R
505	hTLR7_S504	Potential ligand binding residue	uniformly uncharged NH_2 N
527	hTLR7_G526	Potential ligand binding residue	uniformly tiny G
532	hTLR7_Q531	Potential ligand binding residue	uniformly uncharged NH_2 Q
552	hTLR7_N551	Potential ligand binding residue	uniformly uncharged NH_2 N
554	hTLR7_R553	Potential ligand binding residue	uniformly positively charged R
557	hTLR7_L556	Potential ligand binding residue	uniformly aliphatic hydrophobic L
576	hTLR7_S575	Potential ligand binding residue	uniformly small uncharged hydrophilic S
579	hTLR7_H578	Potential ligand binding residue	uniformly positively charged aromatic H

FIGURES

Figure S1. Protein structure of TLR4 (a, c) and TLR7 (b, d) identified by SMART (http://smart.embl-heidelberg.de/) (a, b) and CONSURF (c, d). SMART (a, b) identified following types of domains: **LRR** - Leucine rich repeat; **LRRCT** - Leucine rich repeat C-terminal domain; **TIR** - TIR domain, **Fulfilled blue box (TD)** - transmembrane domain; **LRRNT** - Leucine rich repeat N-terminal domain. **Red box** - LBR (from AA248 to AA469 for TLR4 and from AA495 to AA597 for TLR7). **ECD** - extracellular domain is represented by solid black double arrow; **ICD** - intracellular domain is represented by dashed double arrow. Distal part of ICD (**ICD-DP**) is indicated by a simple solid arrow. Positions of forward and reverse primers used for amplification are shown by arrows. Arrows of same color indicates primer pairs. Description of crystallographic structure (c, d) **LBR** is represented by red polygon; **TD** is present between two dashed lines. To the right from TD is **ICD**, to the left is **ECD**.

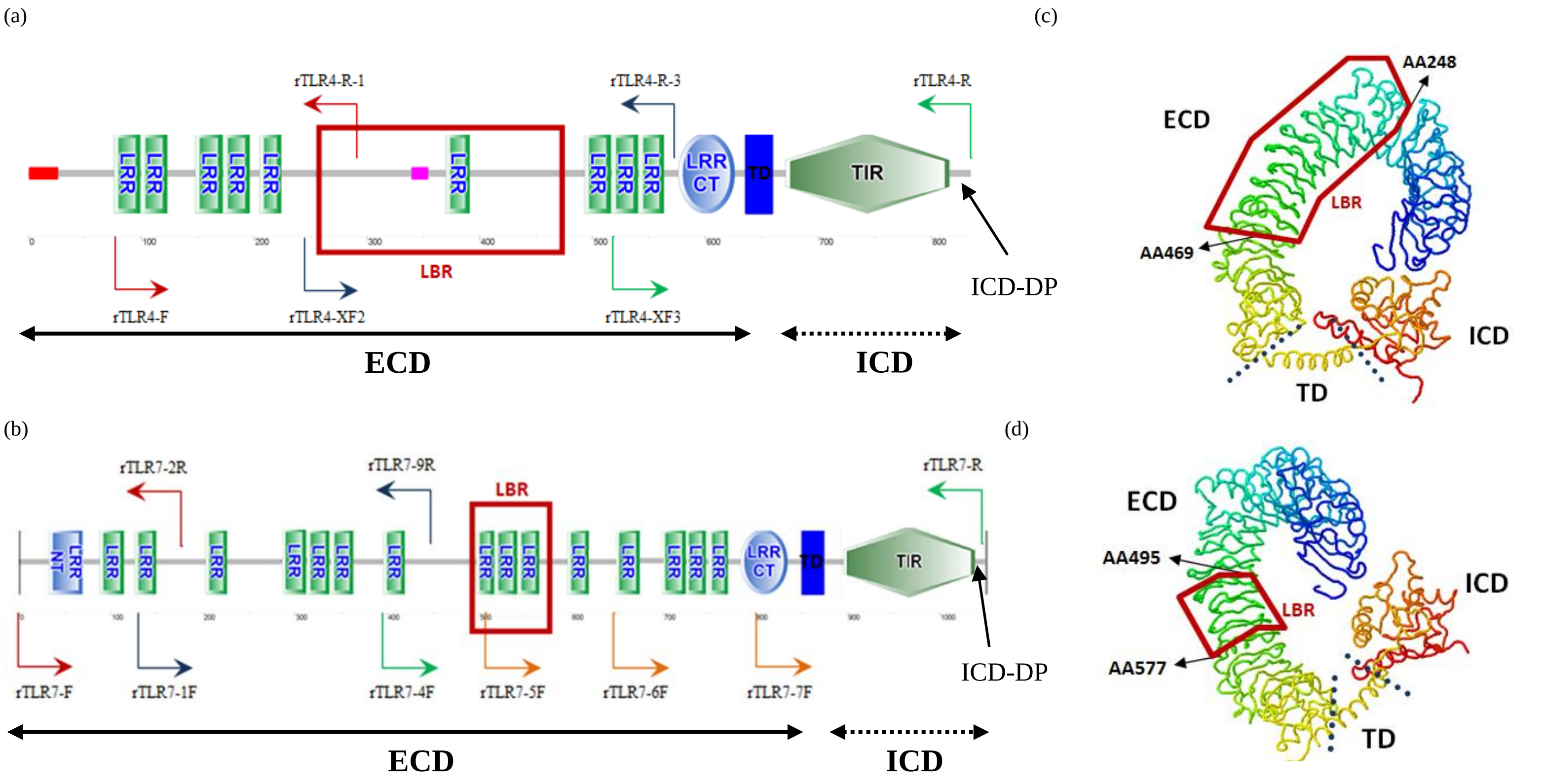

Figures S2 and S3 (Phylogenetic trees) are placed in Additional files 2

Figures S4. Test of congruence between the presumably neutral and *Tlr* phylogenies (*Tlr*4 (a), *Tlr*7 (b) following JANE 4). Number at X axis represents costs of co-divergence. The red dashed line represents the cost observed in our data. The blue columns represent the random distributions of costs. Lower cost than random observed in our data signified higher congruence between species and gene topologies.

(a)

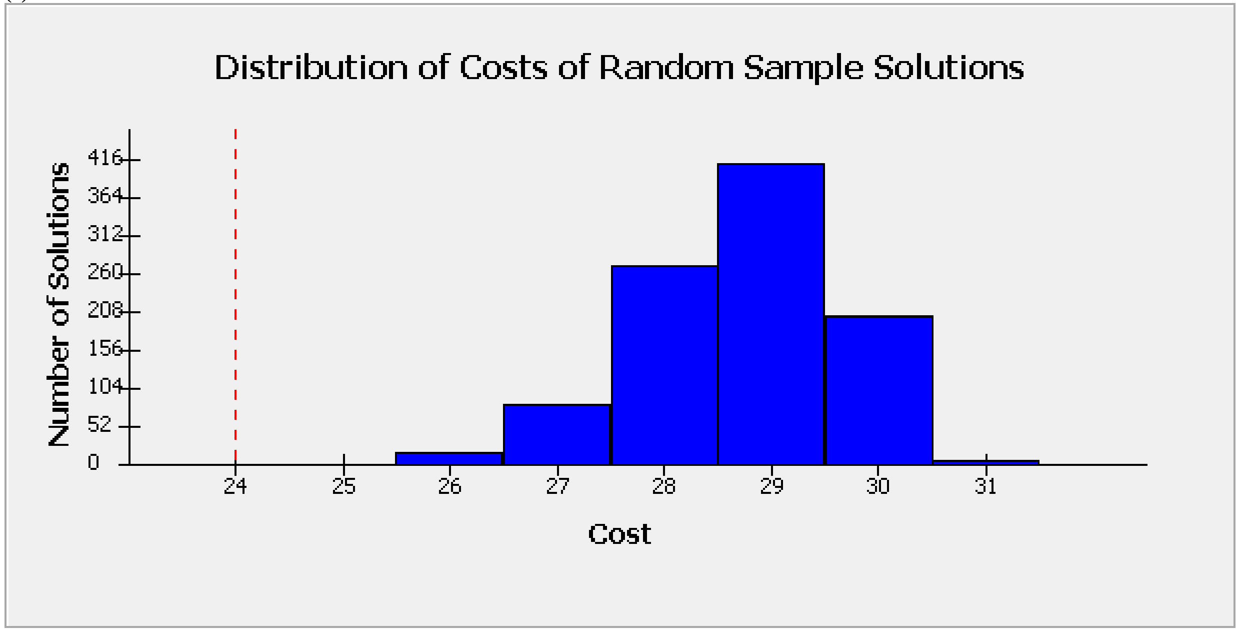

(b)

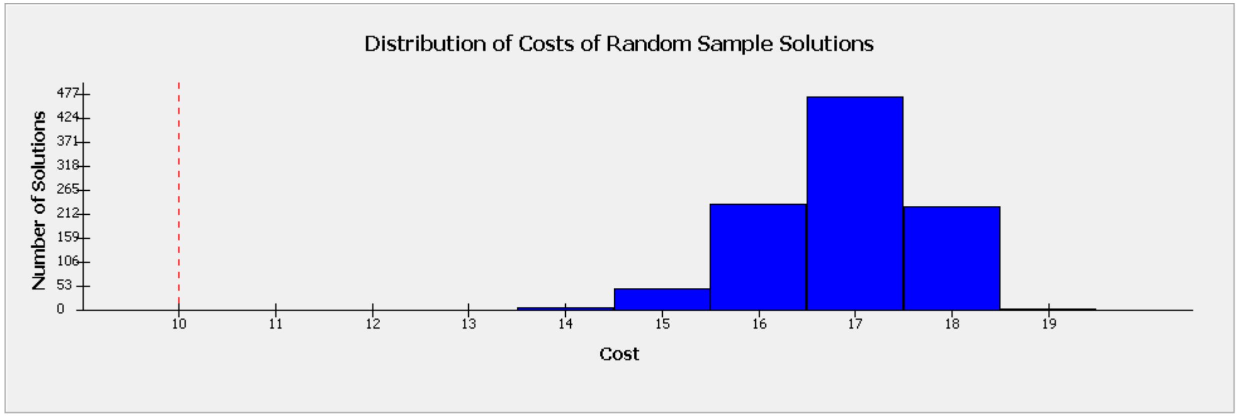

Figure S5. Superimposition of structures, tree clustering diagrams based on linkage distance, (a) LBR_{TLR4} and (b) LBR_{TLR7}; individual LBR-variants often unify more species; description of LBR-variants labels is in the Table S1 under Hap_LBR_{TLR4} and Hap_LBR_{TLR7}.

(a)

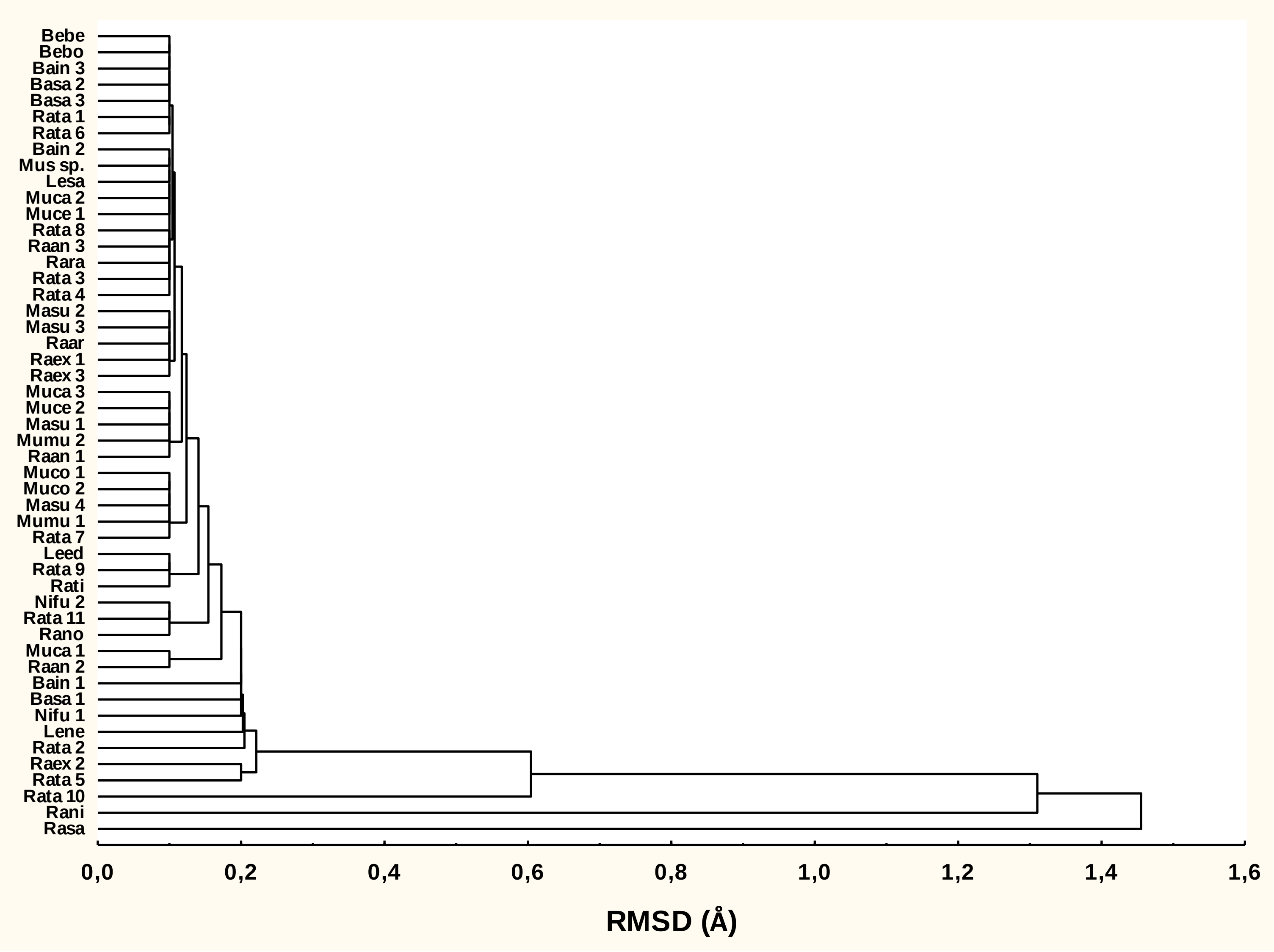

(b)

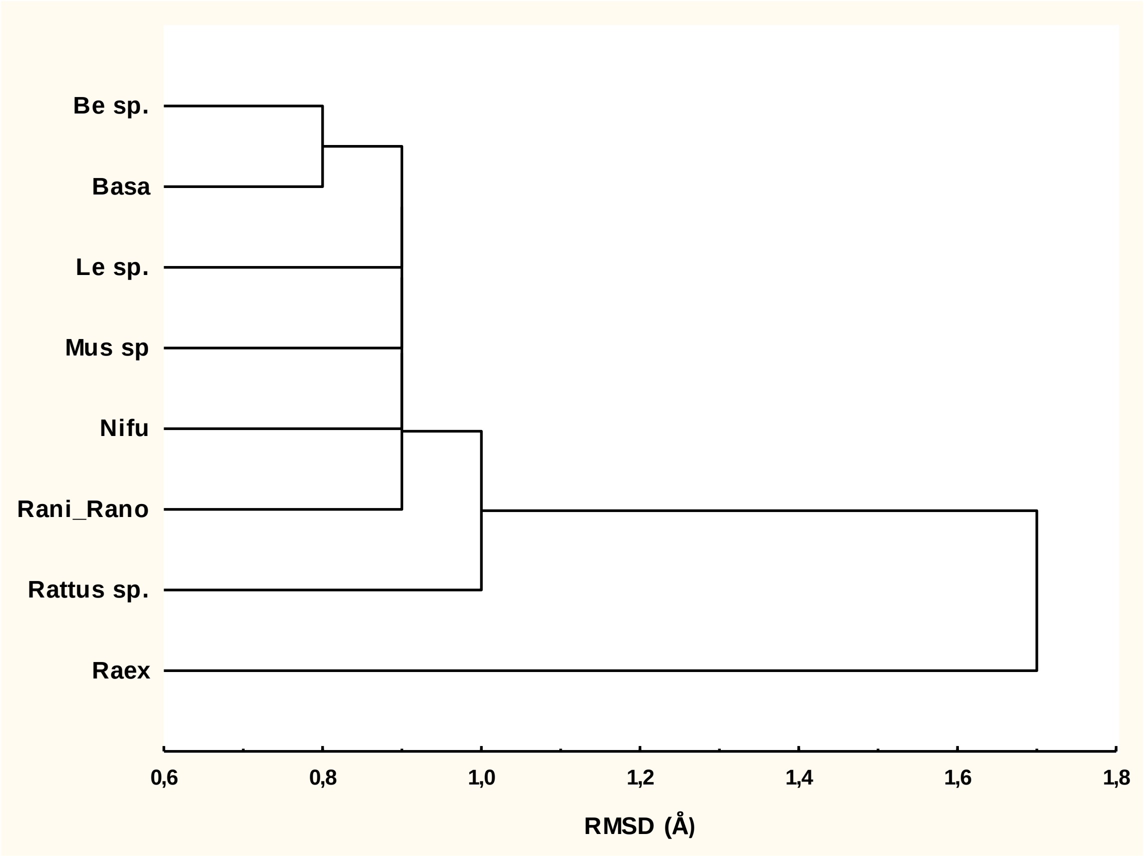
Be sp.
Basa
Le sp.
Mus sp
Nifu
Rani_Rano
Rattus sp.
Raex
0,6
0,8
1,0
1,2
1,4
1,6
1,8
RMSD (Å)

Figure S6. Analysis of LBR amino acid sequence charge at pH 7 (LRRFinder) for (a) LBR_{TLR4} and (b) LBR_{TLR7}, individual LBR-variants often unify more species; description of LBR-variants labels is in the Table S1 under Hap_LBR_{TLR4} and Hap_LBR_{TLR7}. Mouse species are in red, *Rattus* spp. and related genera are in blue.

(a)

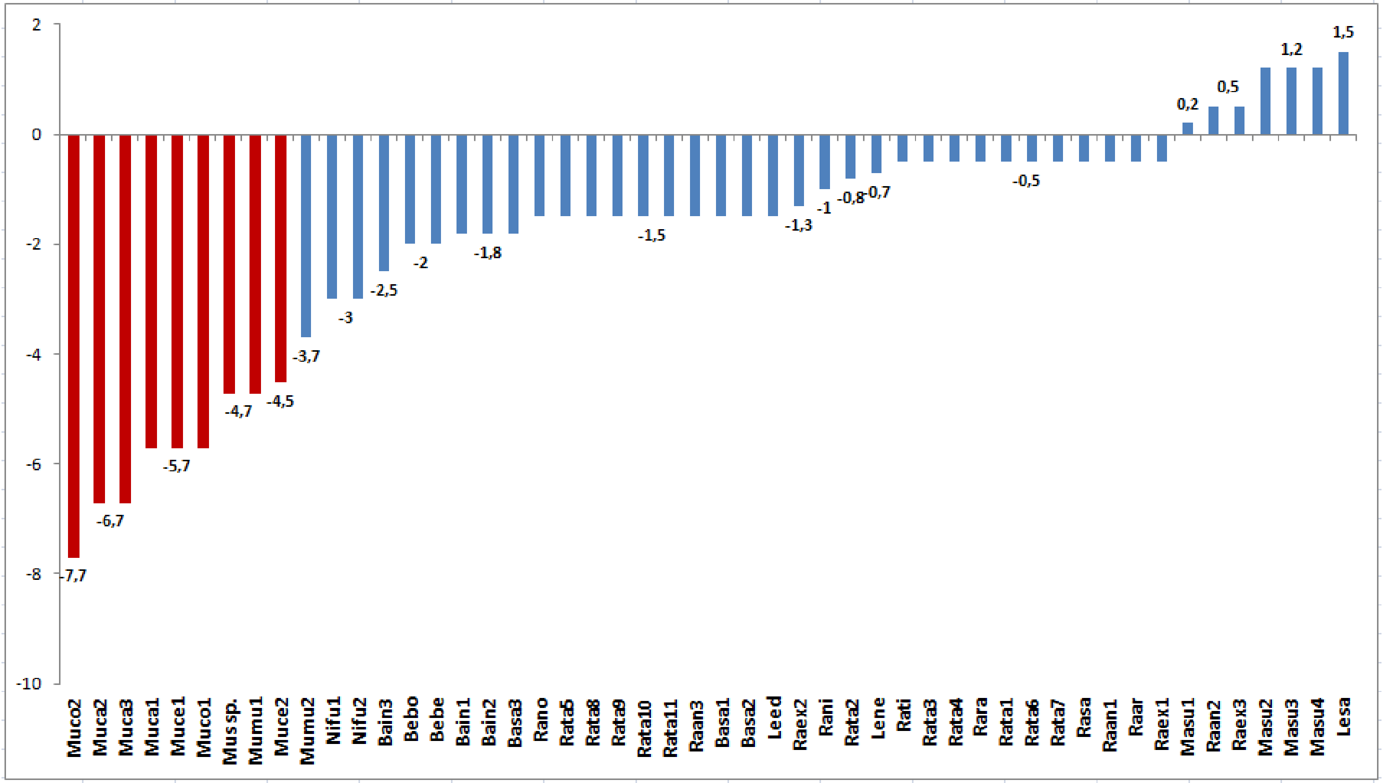

(b)

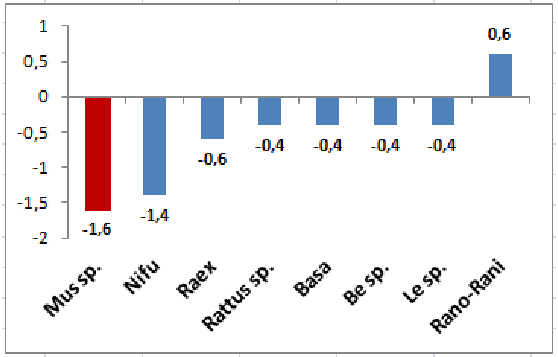

Supplement: Additional file 1: Table S1 — Summary of sampled specimens and identification of haplotypes. Table S2. Primer description. Table S3. Residues binding to LPS in TLR4 based on knowledge of 3D-crystalography in human predicted by Park et al. 2009. Table S4. Potential residues binding ssRNA predicted by Wei et al. 2009. Figure S1. Protein structure of TLR4 (a, c) and TLR7 (b, d) identified by SMART (http://smart.embl-heidelberg.de/) (a, b) and CONSURF (c, d). SMART (a, b) identified following types of domains: LRR - Leucine rich repeat; LRRCT - Leucine rich repeat C-terminal domain; TIR - TIR domain, Fulfilled blue box (TD) - transmembrane domain; LRRNT - Leucine rich repeat N-terminal domain. Red box - LBR (from AA248 to AA469 for TLR4 and from AA495 to AA597 for TLR7). ECD - extracellular domain is represented by solid black double arrow; ICD - intracellular domain is represented by dashed double arrow. Distal part of ICD (ICD-DP) is indicated by a simple solid arrow. Positions of forward and reverse primers used for amplification are shown by arrows. Arrows of same color indicates primer pairs. Description of crystallographic structure (c, d) LBR is represented by red polygon; TD is present between two dashed lines. To the right from TD is ICD, to the left is ECD. Figures S4. Test of congruence between the presumably neutral and Tlr phylogenies (Tlr4 (a), Tlr7 (b) following JANE 4). Number at X axis represents costs of co-divergence. The red dashed line represents the cost observed in our data. The blue columns represent the random distributions of costs. Lower cost than random observed in our data signified higher congruence between species and gene topologies. Figure S5. Superimposition of structures, tree clustering diagrams based on linkage distance, (a) LBRTLR4 and (b) LBRTLR7; individual LBR-variants often unify more species; description of LBR-variants labels is in the Table S1 under Hap_LBRTLR4 and Hap_LBRTLR7. Figure S6. Analysis of LBR amino acid sequence charge at pH 7 (LRRFinder) for [file 1471-2148-13-194-S1.pdf]
